# Phantom evaluation of small‐pixel effect in ultra‐high‐resolution photon‐counting CT: Noise texture and high‐contrast spatial resolution

**DOI:** 10.1002/acm2.70776

**Published:** 2026-08-28

**Authors:** Kyu‐Ho Song, Colin Shan, Gary Xu, Xinhui Duan, Yue Zhang, Kuan Zhang, Liqiang Ren

**Affiliations:** ^1^ Department of Radiology UT Southwestern Medical Center Dallas Texas USA; ^2^ Imaging Services UT Southwestern Medical Center Dallas Texas USA

**Keywords:** modulation transfer function, noise power spectrum, photon‐counting computed tomography, small‐pixel effect, ultra‐high resolution

## Abstract

**Background:**

Ultra‐high‐resolution (UHR) photon‐counting CT (PCCT) with small‐pixel detectors improves resolution and noise, but its dependence on diverse imaging conditions remains insufficiently characterized.

**Purpose:**

To systematically evaluate the small‐pixel effect in UHR PCCT by assessing noise properties and high‐contrast spatial resolution under varying acquisition and reconstruction conditions.

**Methods:**

Images of an ACR CT accreditation phantom with a body ring simulating a medium‐sized patient were acquired on a clinical PCCT system (NAEOTOM Alpha.Peak, Siemens Healthineers, Germany) using standard‐resolution (STD) and UHR modes. Scans were performed at dose levels ranging from 3 to 24 mGy. Reconstructions included slice thicknesses from 0.2/0.4 to 3.0 mm, convolution kernels from Br36 to Br72, quantum iterative reconstruction both off and at strength levels 1–4, matrix sizes of 512 to 1024, and field‐of‐view (FOV) settings from 210 to 350 mm. Both low‐energy threshold (T3D) and virtual monoenergetic images were evaluated. Image noise was quantified using noise magnitude, noise power spectrum (NPS), and average spatial frequency. High‐contrast spatial resolution was evaluated using the modulation transfer function and visual assessment of resolution patterns.

**Results:**

Compared with the STD mode, the UHR mode consistently reduced image noise, with the greatest reductions observed at thinner slice thicknesses and with sharper kernels. In contrast, dose level, QIR strength, matrix size, and spectral image type had minimal influence on the relative noise differences between the two modes. NPS analysis further demonstrated lower noise magnitude and, depending on the imaging conditions, a shift of the noise spectrum toward lower spatial frequencies, indicating relative suppression of high‐frequency noise in the UHR mode. High‐contrast spatial resolution was more strongly influenced by imaging sampling conditions, particularly matrix size and FOV, and remained overall comparable between the STD and UHR modes.

**Conclusions:**

The UHR mode of PCCT consistently achieves lower image noise than the STD mode without compromising high‐contrast spatial resolution. Its greatest benefit is observed in high‐spatial‐resolution imaging protocols employing thin slice thicknesses and sharp reconstruction kernels, whereas the incremental benefit is limited for routine CT imaging. These findings support a task‐based implementation of the UHR mode to optimize protocol selection according to clinical imaging requirements.

## 1 INTRODUCTION

Photon‐counting computed tomography (PCCT) introduces a new standard in medical imaging, offering significant advancements in low‐contrast and high‐contrast resolution, noise reduction, and spectral capabilities compared to traditional energy integrating detector CT (EID‐CT).^1^, ^2^, ^3^ EID‐CT integrates the total energy of incoming X‐rays, while PCCT counts and differentiates individual photons by their energy levels.^2^ This approach provides enhanced material decomposition, mitigates beam‐hardening artifacts,^3^ and optimizes low‐dose imaging performance,^4^ establishing PCCT as an advanced imaging modality.^5^

Among the advantages of PCCT, ultra‐high‐resolution (UHR) imaging leverages smaller detector pixel dimensions to enhance spatial resolution and delineate fine anatomical structures.^1^, ^6^, ^7^ In doing so, the UHR mode inherently exhibits the “small‐pixel effect”, a phenomenon in which systems with smaller detector pixels produce lower image noise than those with larger pixels, under matched reconstruction settings. Although smaller detector pixels receive fewer photons per pixel under identical irradiation conditions, the higher sampling frequency increases the spatial sampling of projection data. When images are reconstructed to a matched spatial resolution, this oversampling allows more effective averaging of quantum noise, resulting in lower image noise compared with larger detector pixels reconstructed at the same resolution.^6^, ^8^ Thus, supported by both theoretical and experimental studies, the small‐pixel effect suggests that UHR acquisitions provide a noise advantage and potential for dose reduction, regardless of whether high spatial resolution is ultimately required.^6^, ^7^, ^9^, ^10^ However, the practical advantages of UHR imaging may depend on system‐related factors such as photon statistics, detector limitations, and reconstruction constraints, which may limit the benefits of UHR acquisitions in certain cases.^6^, ^8^

With the introduction of the first clinical whole‐body PCCT system in 2021, interest in evaluating the small‐pixel effect has been renewed. Recent cadaver studies have explored the dose‐saving potential of UHR mode for various clinical applications, including lumbar spine imaging, CT angiography (CTA) of the lower extremities, femoral arteries, and lungs.^4^, ^11^, ^12^, ^13^ While these studies show promising results—demonstrating reduced image noise and improved contrast‐to‐noise ratios (CNR)—the findings are highly dependent on task‐specific factors such as dose levels, slice thickness, reconstruction kernels, use of filtered back‐projection (FBP) versus iterative reconstruction (IR), and the choice between threshold‐based imaging and virtual monoenergetic imaging (VMI). As a result, the dose reduction potential of UHR mode remains highly task‐dependent and may vary substantially across different clinical acquisition and reconstruction settings, which has been actively explored.

Multiple phantom studies, conducted on both research prototypes and clinical PCCT systems, have investigated the small‐pixel effect and the dose reduction potential of UHR mode. One early study using a research prototype demonstrated that reconstructing UHR data below the system's resolution limit improved dose‐normalized contrast‐to‐noise ratio by up to 34%, suggesting dose savings of up to 43% compared to standard acquisitions.^6^ However, this study focused primarily on CNR metrics with limited scope of reconstruction parameters. Another study conducted on a clinical PCCT system evaluated the small‐pixel effect across three phantom sizes and multiple acquisition settings (dose levels, slice thicknesses, and convolution kernels), showing up to a 3.2‐fold noise reduction and nearly 90% dose savings with UHR mode. Yet, this work did not assess noise texture and relied solely on FBP, omitting the clinically preferred iterative reconstruction algorithms, which may influence both spatial resolution and noise characteristics. Moreover, spatial resolution was evaluated only using smooth kernels, potentially underestimating performance degradation with sharper kernels and IR.^8^ A more recent study using a commercial PCCT scanner found that UHR mode reduced noise magnitude and improved noise texture at all dose levels, with estimated dose savings of ∼33% for abdominal lesions and ∼69% for bone lesions.^14^ Nonetheless, the use of a single quantum iterative reconstruction (QIR) level (4), a fixed slice thickness (0.4 mm), the selection of only two kernels, and the reconstruction of VMIs at non‐matched keV levels limit the generalizability of their findings. Additionally, no prior studies have comprehensively characterized spatial resolution across a broad range of reconstruction techniques.

Therefore, the aim of this study is to comprehensively characterize the small‐pixel effect in PCCT using a systematic phantom‐based evaluation across a wide range of clinically relevant acquisition and reconstruction conditions. We investigate a wide range of radiation dose levels; slice thicknesses spanning from the minimum achievable to the maximum used clinically, reconstruction kernels ranging from smooth to sharp; matrix sizes from 512 to 1024; IR strengths from OFF to 4. Imaging metrics including noise magnitude and texture, and high‐contrast spatial resolution are comprehensively evaluated to assess the performance and clinical implications of the UHR mode in PCCT.

## 2 MATERIALS AND METHODS

### 2.1 Phantom

**FIGURE 1:**
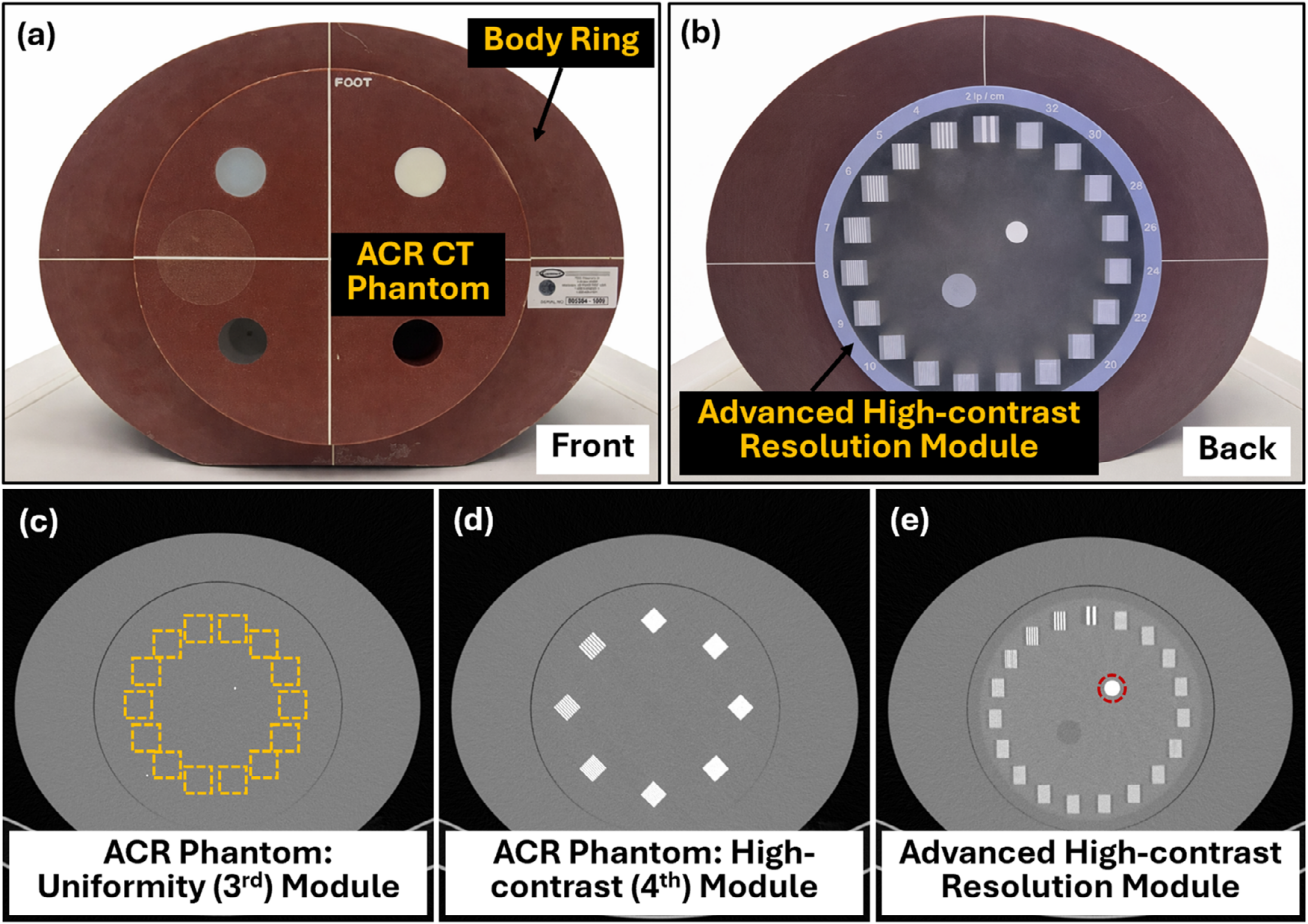
Phantom configuration and representative CT images for image quality assessment. (a) ACR CT accreditation phantom and (b) advanced high‐contrast resolution module positioned within an extension (body) ring to simulate a medium‐sized patient. (c) ROI placements for noise magnitude/texture measurements. (d) High‐contrast resolution patterns ranging from 0.4 to 1.2 lp/mm. (e) Additional high‐contrast patterns up to 3.2 lp/mm. A circular ROI enclosing the high‐contrast insert is indicated for MTF measurements.

An American College of Radiology (ACR) CT accreditation phantom (CTAP: Model 464, Sun Nuclear; 20 × 20 × 16 cm) and an advanced cylindrical high‐contrast resolution module (Advanced iqModules™, Sun Nuclear; 20 × 20 × 4 cm) were stacked longitudinally and placed within an extension ring (oval cross‐section: 33 × 26 cm), simulating attenuation conditions corresponding to a medium‐sized patient, as shown in Figure 1a and b.

The ACR CTAP consists of four modules, of which modules 3 (Figure 1c) and 4 (Figure 1d) were used to evaluate noise power spectrum (NPS) and high‐contrast spatial resolution, respectively. The advanced high‐contrast module, which includes resolution patterns ranging from 0.2‐ to 3.2‐line pairs per millimeter (lp/mm), was incorporated to overcome the limitation of the ACR CTAP (maximum 1.2 lp/mm), thereby enabling assessment of high‐contrast spatial resolution with sharper kernels. This module contains a solid zinc insert with well‐defined edges, which was used for modulation transfer function (MTF) analysis (Figure 1e).

### 2.2 PCCT data acquisition and reconstruction protocol

All scans were performed on a clinical PCCT system (NAEOTOM Alpha.Peak, Siemens Healthineers GmbH, Forchheim, Germany; software version: syngo VB20_SP1) using a routine chest protocol (140 kV, pitch 0.6, and rotation time 0.25 s). Both standard‐resolution (STD; collimation: 144 × 0.4 mm) and UHR (collimation: 120 × 0.2 mm) acquisition modes were evaluated.

The phantom was initially scanned at two radiation dose levels with volume CT dose index (CTDI_vol_) of 3 and 12 mGy, matched between two acquisition modes, representing low‐dose and routine‐dose conditions. Images were reconstructed as low‐energy threshold images (referred to as “T3D” on the scanner), using body regular kernels (Br44 and Br64), quantum iterative reconstruction at strength level 3 (QIR‐3), slice thicknesses of 0.4 and 3.0 mm, and matrix size of 512 (referred to as M512).

To assess dose dependence, extra scans were acquired at CTDI_vol_ of 6 and 24 mGy, representing medium and high‐dose conditions. For the 3‐ and 12‐mGy datasets, additional reconstructions were performed to evaluate the impact of key imaging parameters:

a. slice thickness: 0.4 to 3.0 mm (STD) and 0.2 to 3.0 mm (UHR), with M512, QIR‐3, and Br44/Br64;
b. convolutional kernel: Br36 to Br72, with 0.4/3.0 mm, M512, and QIR‐3;
c. QIR level: QIR‐OFF and 1 to 4, with 0.4/3.0 mm, M512, and Br44/Br64;
d. matrix size: 512 to 1024, with 0.4/3.0 mm, QIR‐3, and Br44/Br64;
e. VMI: 70 keV, with 0.4/3.0 mm, M512, QIR‐3, and Br44/Br64; and
f1. reconstruction field‐of‐view (rFOV) for noise evaluation: 350 mm for all images reconstructed with the Br44 kernel, and 210 mm for images reconstructed with the Br64 kernel and those used for kernel dependence evaluation. The rationale for selecting kernel‐dependent rFOV is described in the following section.
f2. rFOV for spatial resolution evaluation: 210 to 350 mm, with 0.4/3.0 mm, QIR‐3, M512, Br44/Br64.

All acquisitions and reconstructions were repeated three times, with detailed parameters summarized in Table 1.

**TABLE 1:** Summary of essential PCCT data acquisition and image parameters.

	Parameter	Values	Fixed Parameters	Representative figures
**Acquisition**	Mode	STD, UHR	‐	‐
	CTDI_vol_ (mGy)	3, 6, 12, 24	‐	Primary: Figs. 2, 3; Additional: Figs. S1, S2
	Focal spot (mm)	STD: 0.8 × 1.1 (all dose levels) UHR: 0.4 × 0.4 (3/6 mGy); 0.6 × 0.7 (12 mGy); 0.8 × 1.1 (24 mGy);	‐	‐
**Baseline Reconstruction**	Image type	T3D	‐	‐
	Slice thickness (mm)	0.4, 3.0	‐	‐
	Matrix size	512 (M512)	‐	‐
	QIR strength	3	‐	‐
	Kernel	Br44, Br64	‐	‐
	Field of view (FOV: mm)	350 (Br44), 210 (Br64) 210 (all other kernels)	‐	‐
**Reconstruction parameter variations (3 and 12 mGy only)**	Slice thickness (mm)	STD: 0.4 ‐ 3.0; UHR: 0.2 ‐ 3.0	T3D, M512, QIR‐3, Br44/Br64	Figs. 4, 5; Figs. S3, S4
	Kernel	Br36 ‐ Br72	T3D, 0.4/3.0 mm, M512, QIR‐3	Figs. 6, 7; Figs. S5–S7
	QIR strength	Off, 1 ‐ 4	T3D, 0.4/3.0 mm, M512, Br44/Br64	Figs. 8, 9; Figs. S8–S11
	Matrix size	512 ‐ 1024	T3D, 0.4/3.0 mm, QIR‐3, Br44/Br64	Figs. 10, 11; Figs. S12–S15
	Image type	T3D vs. VMI (70 keV)	0.4/3.0 mm, M512, QIR‐3, Br44/Br64	Figs. 12, 13; Fig. S16, S17
	FOV (mm)	210 ‐ 350	T3D, 0.4/3.0 mm, M512, QIR‐3, Br44/Br64	Fig. 14; Fig. S18

### 2.3 Image quality analysis

#### 2.3.1 Noise magnitude and texture

The NPS was computed using a web‐based platform for CT image quality evaluation and radiation dose optimization (CTPro: http://www.ctpro.net) to characterize noise magnitude and texture.^15^ As shown in Figure 1c, 2D NPS curves were generated by placing square regions of interest (ROIs) in a circular arrangement within the CTAP's uniform module, ensuring that ROIs did not overlap or contact adjacent boundaries. The number of central slices included in the analysis was determined based on slice thickness and increment to fully utilize the central ∼3.0 cm of the module. Square ROIs of approximately 16.4 × 16.4 mm^2^ were used for NPS analysis. ROI size, positions, and the selected slice range were kept consistent between acquisition modes (STD vs. UHR) to enable direct comparison.

**FIGURE 2:**
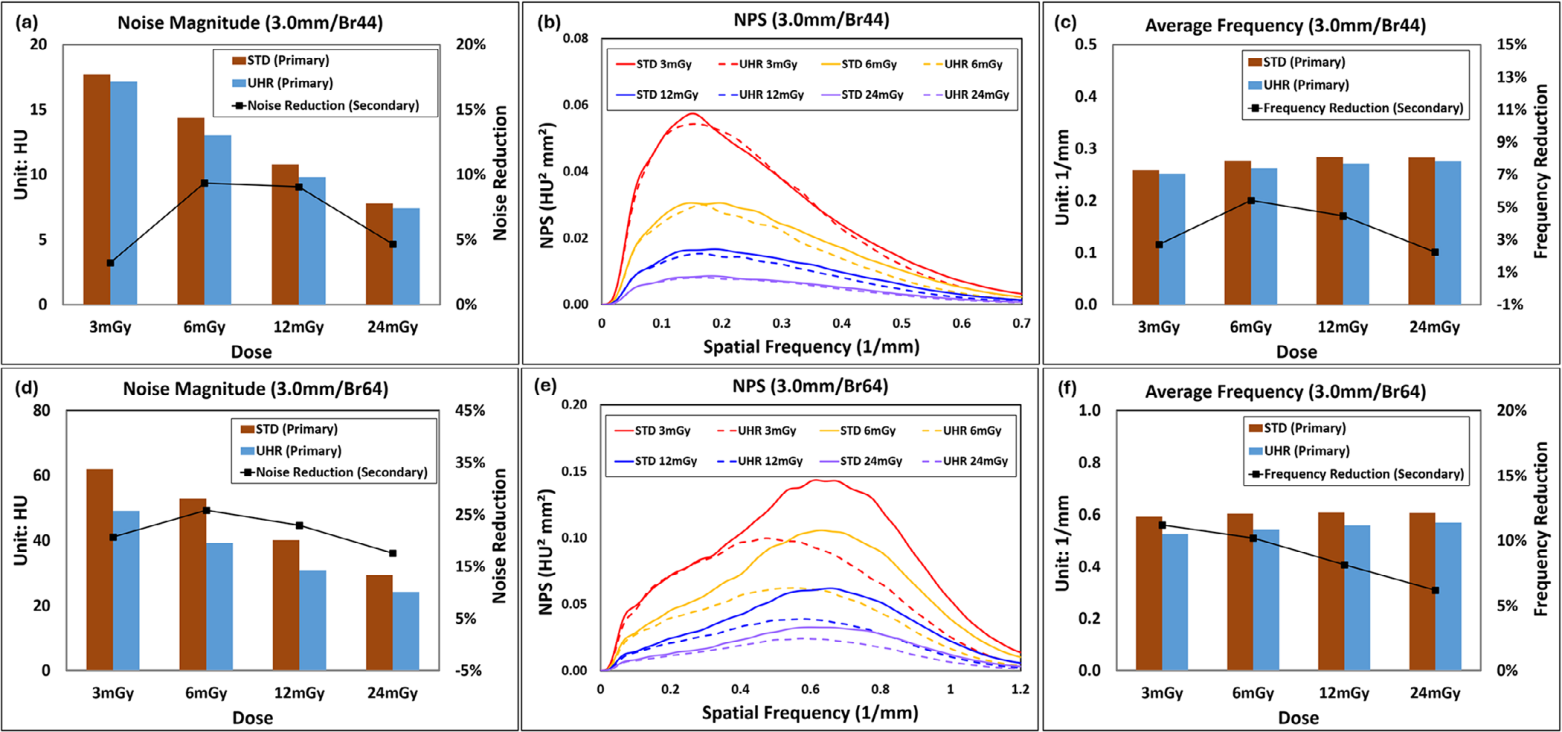
Effect of radiation dose on noise magnitude, NPS, and average spatial frequency from T3D images for STD and UHR modes (3 mm slice thickness). Results are shown for Br44 (a—c) and Br64 (d—f): noise magnitude (a, d), NPS (b, e), and average spatial frequency (c, f), with percentage reduction on the secondary axis. Note: The standard deviation was negligible (SD < 0.3 HU for noise magnitude and SD < 0.002 mm^−^^1^ for average spatial frequency).

The reconstruction FOV was selected to ensure adequate spatial frequency coverage for NPS characterization, particularly at higher frequencies. For example, with a 350 mm FOV and a 512 × 512 matrix, the pixel size is 0.684 mm, corresponding to a Nyquist frequency of 0.73 $mm^{-1}$, which is sufficient for smoother kernels (e.g., Br44), but not for sharper kernels (e.g., Br64). Therefore, a smaller FOV of 210 mm was used for Br64 kernels, yielding a Nyquist frequency of 1.22 $mm^{-1}$.

Noise magnitude was quantified as the square root of the area under the NPS curve. To further characterize noise texture, the average spatial frequency ($f_{avg}$) was calculated from the NPS to represent the dominant frequency content of image noise:

(1) $$f_{avg}=\frac{\int_{0}^{f_{max}}f\cdot NPS\left(f\right)df}{\int_{0}^{f_{max}}NPS\left(f\right)df}$$

where $f_{max}$ is the maximum spatial frequency and NPS(f) is the NPS at frequency *f*.^16^

#### 2.3.2 High‐contrast spatial resolution

To evaluate high‐contrast spatial resolution, all patterns within both high‐contrast modules were visually compared (Figure 1d and 1e). In addition, MTF analysis was performed by placing a circular ROI over the solid zinc insert (Figure 1e). This selected insert has a well‐defined sharp edge, which was crucial for accurate edge spread function (ESF) and line spread function (LSF) calculation.^17^, ^18^, ^19^ To reduce image noise, selected consecutive slices were averaged prior to analysis. The center of the insert was manually specified, and its radius was automatically refined by identifying the radius that maximized the mean radial edge gradient. An oversampled radial ESF was generated by grouping pixel intensities into sub‐pixel bins according to their signed radial distance from the refined circular edge. To improve the robustness of the ESF against residual image noise while preserving the edge transition, Savitzky–Golay filtering was applied to smooth local intensity variations.^20^, ^21^

The LSF was obtained by differentiating the filtered ESF. After baseline correction, a Tukey window was applied to the LSF prior to Fourier transformation (FT) to reduce spectral leakage.^18^ The MTF was calculated as the normalized magnitude of the FT of the windowed LSF. Spatial resolution was quantified using the spatial frequencies corresponding to the 50% and 10% MTF levels (MTF50 and MTF10). Along with the MTF analysis, high‐contrast patterns were also compared between the STD and UHR modes to qualitatively assess the spatial resolution performance.

## 3 RESULTS

### 3.1 Effect of radiation dose

Compared with the STD mode, the UHR mode consistently reduced image noise across all dose levels by 3–9% for Br44 and 18–26% for Br64 (Figure 2a,d). NPS analysis for Br44 demonstrated a modest reduction in overall noise magnitude with minimal changes in spectral shape (Figure 2b), consistent with only small differences in average spatial frequency (≤5%; Figure 2c). In contrast, Br64 exhibited both a reduction in NPS magnitude and a shift toward lower spatial frequencies (Figure 2e), reflected by a 6–11% decrease in average spatial frequency for UHR (Figure 2f).

**FIGURE 3:**
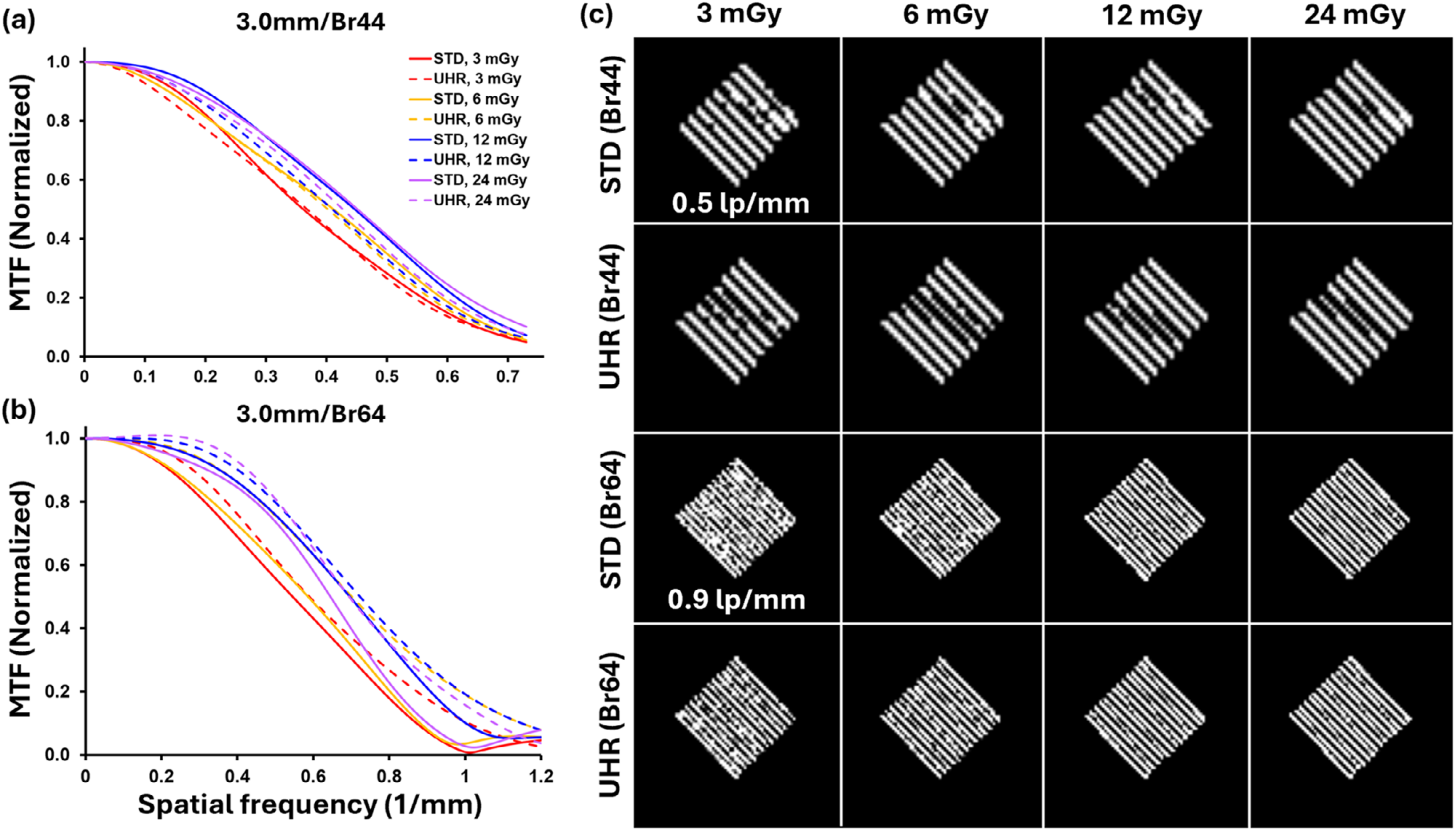
Representative MTF curves measured from T3D images and high‐contrast images for STD and UHR modes at 3.0 mm slice thickness across dose levels (3–24 mGy). Results are shown for Br44 (a) and Br64 (b), with corresponding images of high‐contrast spatial resolution patterns (c).

MTF analysis also demonstrated kernel‐dependent behavior. For Br44 (Figure 3a, Table 2), the UHR mode produced slightly lower spatial resolution than the STD mode, with MTF50 decreasing by approximately 5–7% and MTF10 by 2–5% under most conditions. In contrast, for Br64 (Figure 3b, Table 2), the UHR mode improved spatial resolution, increasing MTF50 by 4–29% and MTF10 by up to 23%, relative to the corresponding STD mode. Representative images of high‐contrast spatial resolution patterns are shown in Figure 3c. Additional results for the thinner slice thickness (0.4 mm) are provided in Figures S1 and S2.

**TABLE 2:** MTF Metrics for STD and UHR Modes Across Dose Levels and Slice Thicknesses.

		STD (1/mm)		UHR (1/mm)		Difference (%)	
		MTF50%	MTF10%	MTF50%	MTF10%	MTF50%	MTF10%
Dose [mGy] (3.0mm/Br44)	3	0.354	0.634	0.373	0.647	5.6	2.0
	6	0.408	0.679	0.397	0.663	−2.8	−2.4
	12	0.438	0.699	0.407	0.667	−7.0	−4.7
	24	0.452	0.722	0.429	0.693	−5.2	−4.0
Dose [mGy] (3.0mm/Br64)	3	0.501	0.839	0.646	1.035	29.1	23.3
	6	0.603	0.898	0.671	1.106	11.3	23.1
	12	0.677	0.971	0.707	1.123	4.3	15.7
	24	0.642	0.927	0.709	1.123	10.4	21.2
Slice thickness [mm] (12mGy/Br44)	0.2	‐	‐	0.419	0.659	‐	‐
	0.4	0.438	0.694	0.413	0.657	−5.8	−5.4
	1.0	0.440	0.700	0.409	0.668	−7.1	−4.6
	3.0	0.438	0.699	0.407	0.667	−7.0	−4.7
Slice thickness [mm] (12mGy/Br64)	0.2	‐	‐	0.756	1.128	‐	‐
	0.4	0.682	0.981	0.735	1.159	7.9	18.2
	1.0	0.677	0.983	0.738	1.160	9.0	17.9
	3.0	0.677	0.971	0.707	1.123	4.3	15.7

Values represent the mean of three repeated measurements.

Difference (%) = (UHR − STD) / STD × 100.

### 3.2 Effect of slice thickness

**FIGURE 4:**
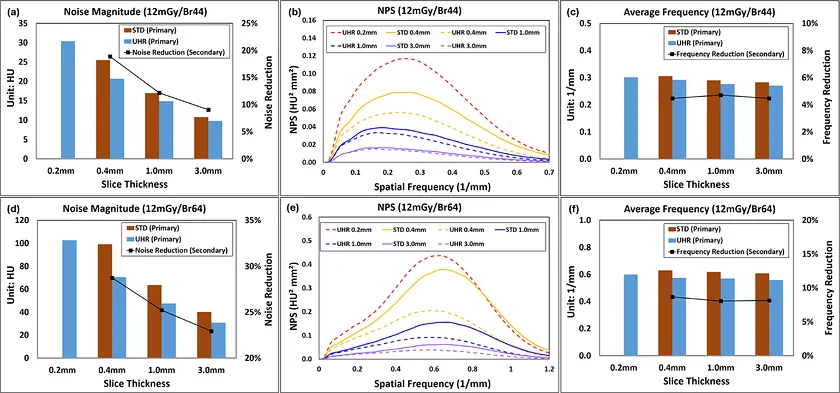
Effect of slice thickness on noise magnitude, NPS, and average spatial frequency measured from T3D images for STD and UHR modes at 12 mGy. Results are shown for Br44 (a–c) and Br64 (d–f): noise magnitude (a, d), NPS (b, e), and average spatial frequency (c, f). Note: The standard deviation was negligible (SD < 0.3 HU for noise magnitude and SD < 0.002 mm^−^^1^ for average spatial frequency).

Compared with the STD mode, the UHR mode consistently reduced image noise across all slice thicknesses by 9–19% for Br44 (Figure 4a) and 23–29% for Br64 (Figure 4d). NPS analysis for both kernels demonstrated greater reductions in overall noise magnitude with the UHR mode at thinner slice thickness while maintaining a similar spectral shape across slice thickness (Figure 4b,e), consistent with only modest changes in average spatial frequency: 4–5% for Br44 (Figure 4c) and 8–9% for Br64 (Figure 4f).

**FIGURE 5:**
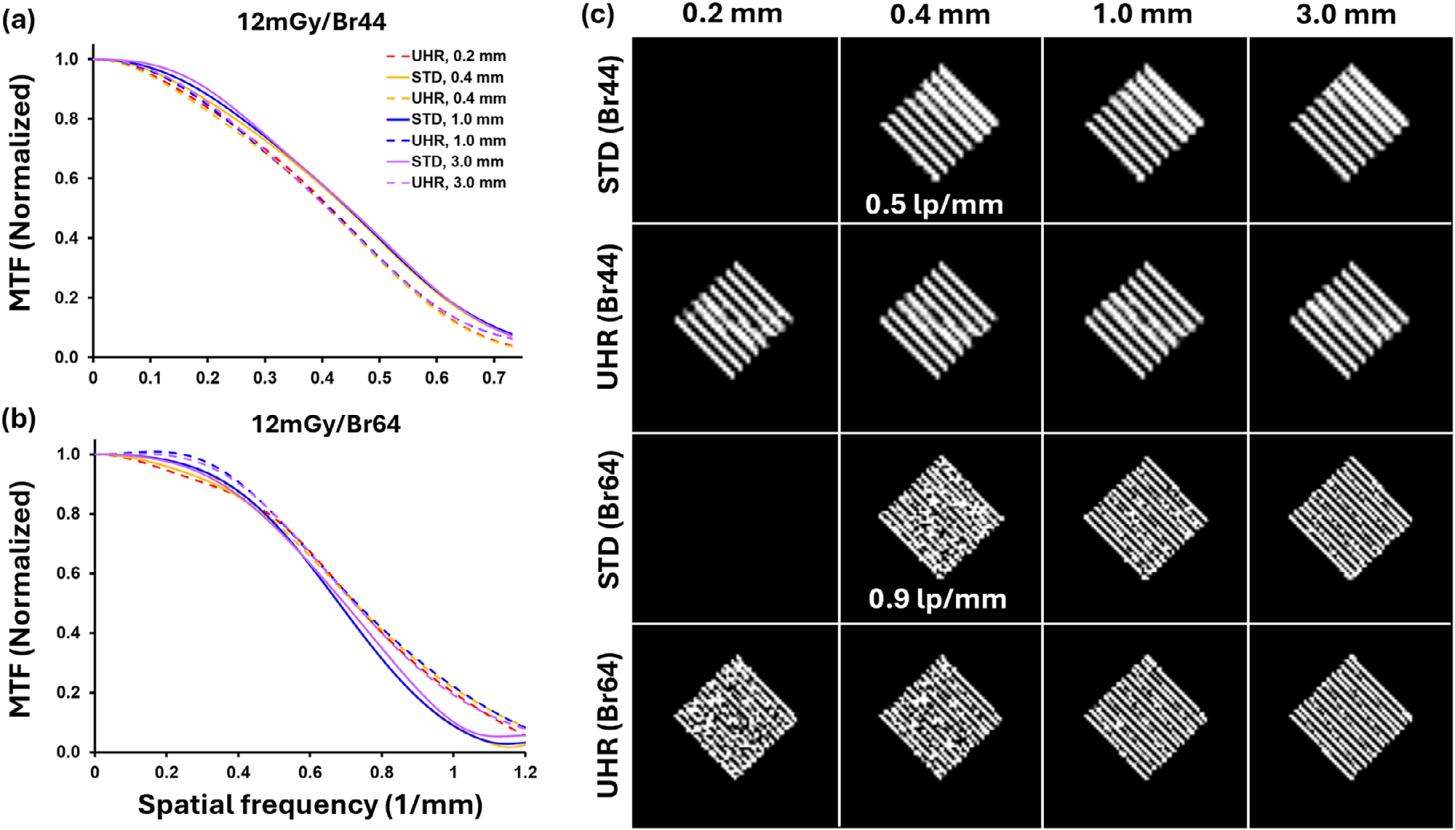
Representative MTF curves measured from T3D images and high‐contrast images for STD and UHR modes at 12 mGy across slice thicknesses (0.2–3.0 mm). Results are shown for Br44 (a) and Br64 (b), with corresponding images of high‐contrast spatial resolution patterns (c).

MTF analysis likewise demonstrated kernel‐dependent behavior. For Br44 (Figure 5a; Table 2), the UHR mode produced slightly lower spatial resolution than the STD mode, with MTF50 decreasing by approximately 6–7% and MTF10 by approximately 5%. In contrast, for Br64 (Figure 5b; Table 2), the UHR mode improved spatial resolution, increasing MTF50 by 4–9% and MTF10 by up to 18%, relative to the corresponding STD mode. Representative images of high‐contrast spatial resolution patterns are shown in Figure 5c. Additional results acquired at a lower dose (3 mGy) are presented in Figures S3 and S4.

### 3.3 Effect of reconstruction kernels

**FIGURE 6:**
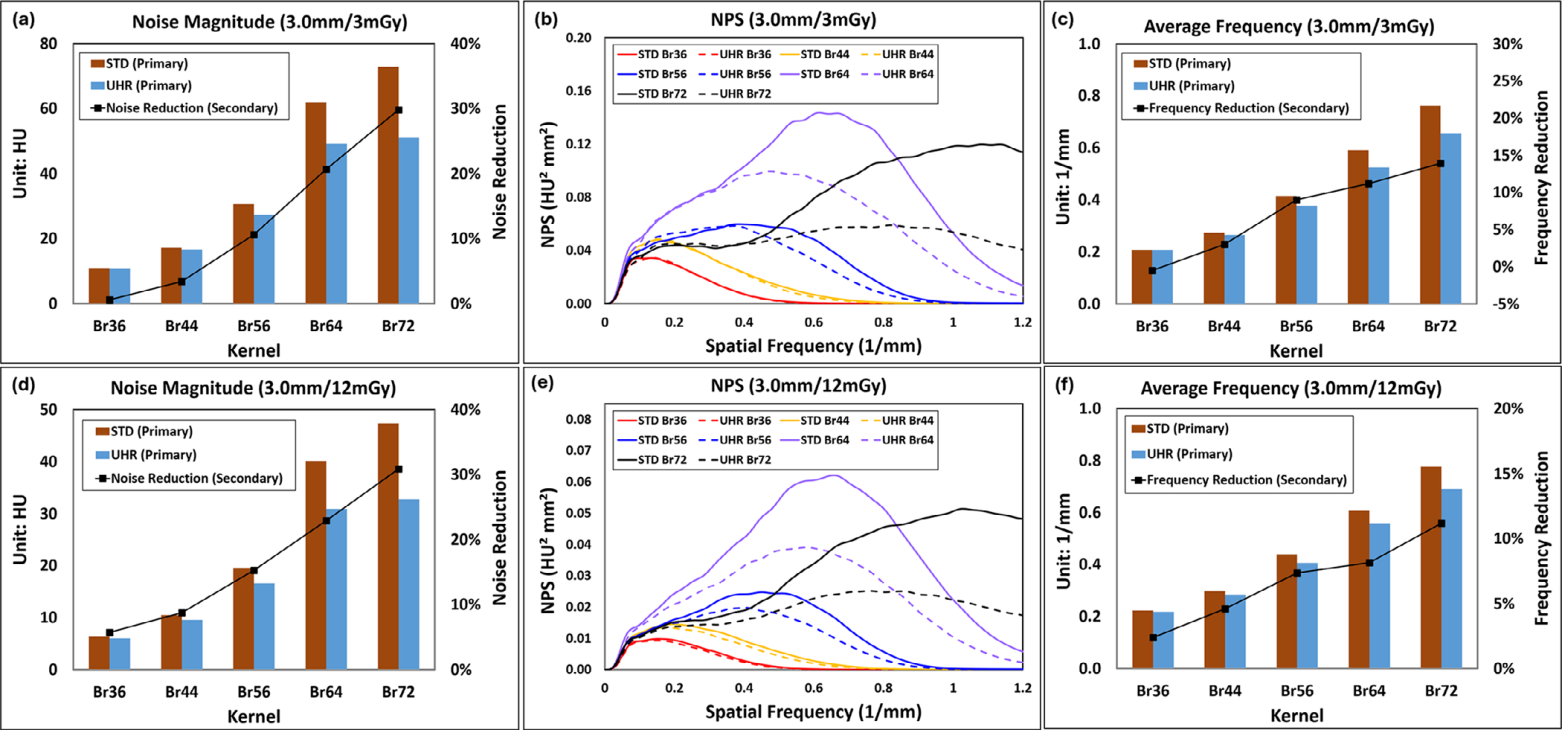
Effect of reconstruction kernel on noise magnitude, NPS, and average spatial frequency measured from T3D images for STD and UHR modes (3.0 mm slice thickness). Results are shown for 3 mGy (a—c) and 12 mGy (d—f): noise magnitude (a, d), NPS (b, e), and average spatial frequency (c, f), with percentage reduction on the secondary axis. Note: The standard deviation was negligible (SD < 0.3 HU for noise magnitude and SD < 0.002 mm^−^^1^ for average spatial frequency).

**FIGURE 7:**
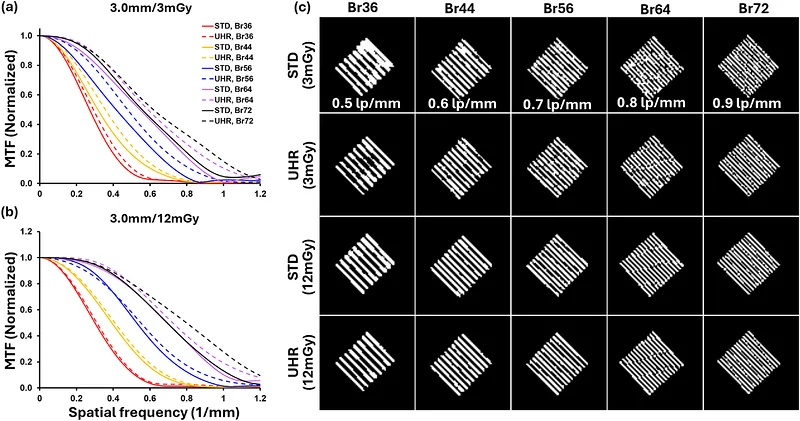
Representative MTF curves measured from T3D images and high‐contrast images for STD and UHR modes at 3.0 mm slice thickness across reconstruction kernels. Results are shown for 3 mGy (a) and 12 mGy (b), with corresponding images of high‐contrast spatial resolution patterns (c).

Compared with the STD mode, the UHR mode consistently reduced image noise across all kernels (Figure 6a,d), with greater reductions observed for sharper kernels (up to 31% for Br72). NPS analysis demonstrated lower NPS magnitude for the UHR mode than for the STD mode, particularly in the high‐frequency region (Figure 6b,e). Consistent with these findings, average spatial frequency increased with kernel sharpness in both modes (Figure 6c,f) but remained up to 14% lower in the UHR mode, indicating relative suppression of high‐frequency noise components. Qualitative comparisons of noise characteristics across kernels are shown in Figure S5. MTF analysis demonstrated consistent improvements in spatial resolution with the UHR mode across all kernels (Figure 7a,b; Table 3), with MTF50 increasing by 10–45% (3 mGy) and 4–13% (12 mGy), and MTF10 by 8–27% (3 mGy) and 3–17% (12 mGy) relative to the corresponding STD mode. Representative high‐contrast spatial resolution patterns are shown in Figure 7c. Additional kernel‐dependent results acquired at thinner slice thickness are presented in Figures S6 and S7.

**TABLE 3:** MTF Metrics for STD and UHR Modes Across Kernels Using a Small FOV.

		STD (1/mm)		UHR (1/mm)		Difference (%)	
		MTF50%	MTF10%	MTF50%	MTF10%	MTF50%	MTF10%
Kernel (3.0mm/3mGy)	Br36	0.274	0.474	0.300	0.513	9.5	8.3
	Br44	0.323	0.608	0.373	0.658	15.7	8.3
	Br56	0.427	0.753	0.511	0.858	19.6	14.0
	Br64	0.501	0.839	0.646	1.035	29.1	23.3
	Br72	0.497	0.857	0.721	1.084	45.1	26.5
Kernel (3.0mm/12mGy)	Br36	0.588	0.884	0.611	0.931	3.8	5.3
	Br44	0.388	0.672	0.409	0.691	5.5	2.9
	Br56	0.542	0.852	0.562	0.912	3.7	7.0
	Br64	0.677	0.971	0.707	1.123	4.3	15.7
	Br72	0.721	1.015	0.816	1.184	13.2	16.6

Values represent the mean of three repeated measurements.

Difference (%) = (UHR − STD) / STD × 100.

### 3.4 Effect of quantum iterative reconstruction

**FIGURE 8:**
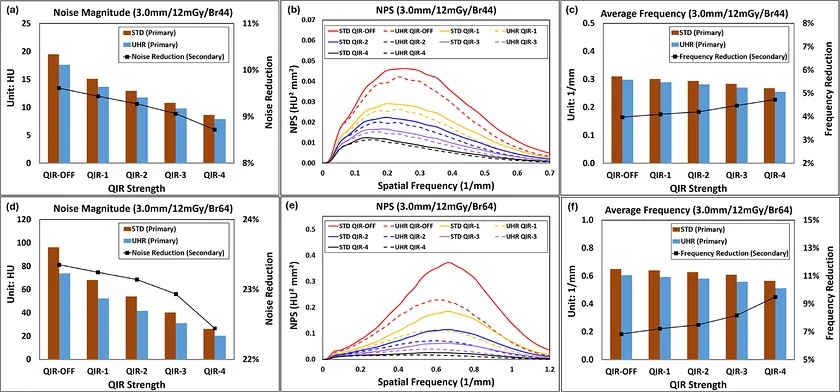
Effect of QIR on noise magnitude, NPS, and average spatial frequency measured from T3D images for STD and UHR modes (3.0 mm slice thickness, 12 mGy). Results are shown for Br44 (a—c) and Br64 (d—f): noise magnitude (a, d), NPS (b, e), and average spatial frequency (c, f), with percentage reduction on the secondary axis. Note: The standard deviation was negligible (SD < 0.3 HU for noise magnitude and SD < 0.002 mm^−^^1^ for average spatial frequency).

Compared with the STD mode, the UHR mode consistently reduced image noise across all QIR strengths by approximately 10% for Br44 (Figure 8a) and 23% for Br64 (Figure 8d). NPS analysis demonstrated lower magnitude for the UHR mode while preserving a similar spectral shape across all QIR strengths for both kernels (Figure 8b,e). Consistent with these findings, average spatial frequency was slightly lower for the UHR mode across all QIR strengths for both kernels (Figure 8c,f).

**FIGURE 9:**
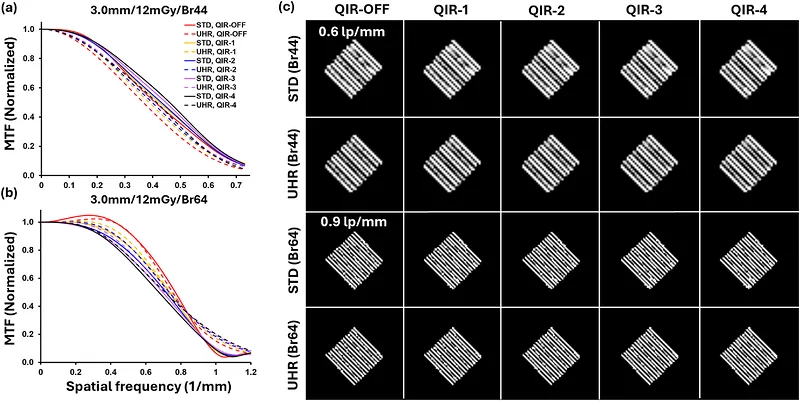
Representative MTF curves measured from T3D images and high‐contrast images for STD and UHR modes (3.0 mm slice thickness, 12 mGy) across QIR levels. Results are shown for Br44 (a) and Br64 (b), with corresponding images of high‐contrast spatial resolution patterns (c).

MTF analysis again demonstrated kernel‐dependent behavior (Figure 9a,b; Table 4). For Br44, the UHR mode exhibited slightly lower spatial resolution than the STD mode, with MTF50 reduced by 6–8% and MTF10 by 5–8%. In contrast, for Br64, the UHR mode increased MTF10 by 15–18% across all QIR strengths, whereas MTF50 was comparable at QIR‐OFF and increased by up to 8% at higher QIR strengths. Representative high‐contrast spatial resolution patterns are shown in Figure 9c. Additional QIR‐dependent results across different dose and slice thickness conditions are presented in Figures S8–S11.

**TABLE 4:** MTF Metrics for STD and UHR Modes Across QIR Levels and Matrix Sizes.

		STD (1/mm)		UHR (1/mm)		Difference (%)	
		MTF50%	MTF10%	MTF50%	MTF10%	MTF50%	MTF10%
QIR (3.0mm/12mGy/Br44)	0	0.399	0.678	0.368	0.634	−7.7	−6.4
	1	0.411	0.712	0.385	0.653	−6.3	−8.3
	2	0.423	0.714	0.396	0.662	−6.4	−7.2
	3	0.438	0.699	0.407	0.667	−7.0	−4.7
	4	0.452	0.707	0.416	0.659	−7.9	−6.8
QIR (3.0mm/12mGy/Br64)	0	0.745	0.952	0.743	1.097	−0.2	15.3
	1	0.712	0.956	0.730	1.110	2.6	16.1
	2	0.694	0.961	0.719	1.117	3.6	16.2
	3	0.677	0.971	0.707	1.123	4.3	15.7
	4	0.647	0.956	0.699	1.132	8.1	18.3
Matrix (3.0mm/12mGy/Br44)	512	0.438	0.699	0.407	0.667	−7.0	−4.7
	768	0.434	0.733	0.432	0.731	−0.6	−0.2
	1024	0.454	0.761	0.442	0.742	−2.7	−2.5
Matrix (3.0mm/12mGy/Br64)	512	0.677	0.971	0.707	1.123	4.3	15.7
	768	0.801	1.182	0.843	1.316	5.2	11.4
	1024	0.821	1.241	0.877	1.357	6.8	9.4

Values represent the mean of three repeated measurements.

Difference (%) = (UHR − STD) / STD × 100.

### 3.5 Effect of matrix size

**FIGURE 10:**
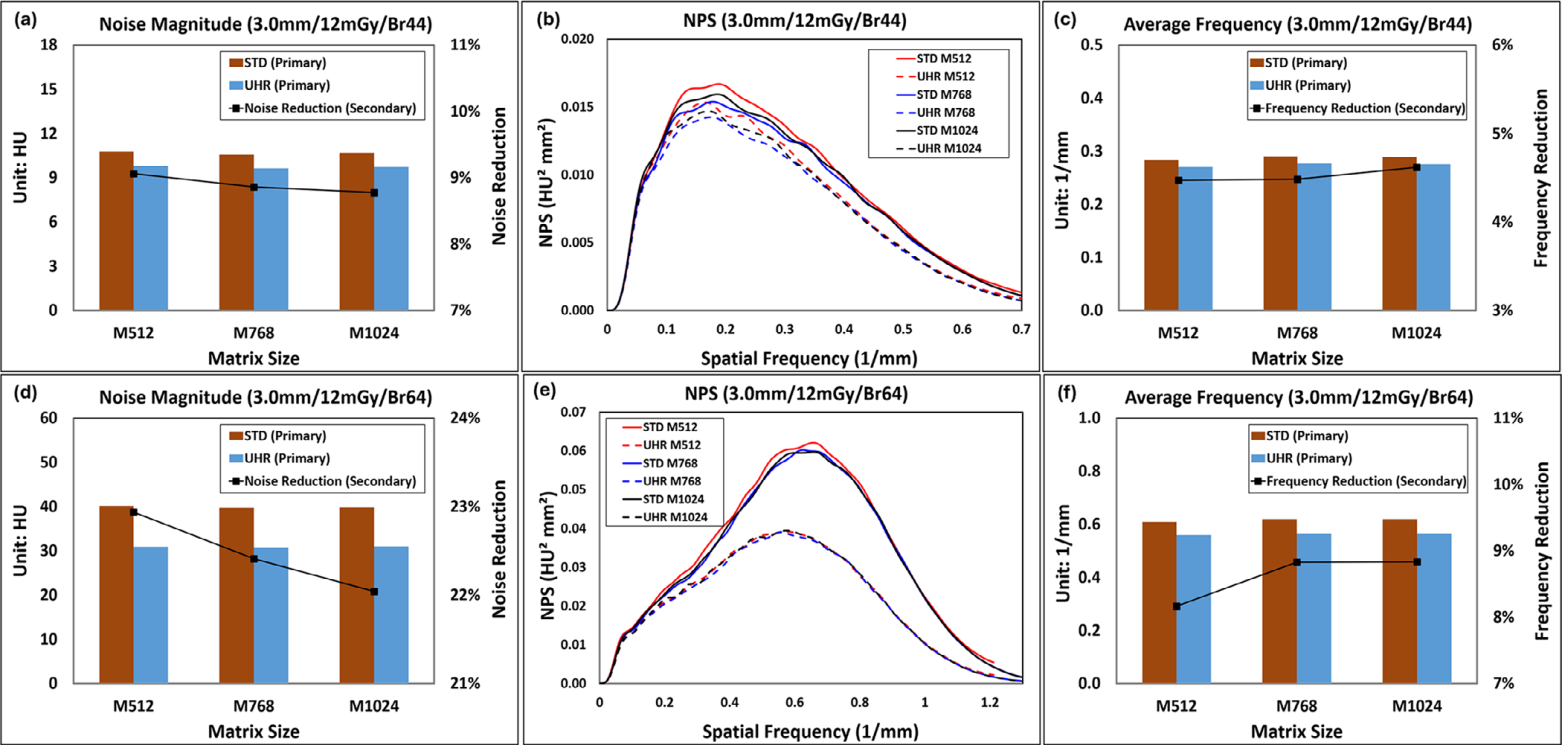
Effect of matrix size on noise magnitude, NPS, and average spatial frequency measured from T3D images for STD and UHR modes (3.0 mm slice thickness, 12 mGy). Results are shown for Br44 (a—c) and Br64 (d—f): noise magnitude (a, d), NPS (b, e), and average spatial frequency (c, f), with percentage reduction on the secondary axis. Note: The standard deviation was negligible (SD < 0.3 HU for noise magnitude and SD < 0.002 mm^−^^1^ for average spatial frequency).

For both Br44 and Br64 kernels, image noise showed minimal dependence on matrix size in both the STD and UHR modes (Figure 10a,d). NPS analysis likewise demonstrated negligible effects of matrix size on noise characteristics (Figure 10b,e), with both noise magnitude and spectral shape remaining consistent across all matrix sizes. Consistent with these findings, average spatial frequency varied only slightly (Figure 10c,f), with small absolute differences and consistent relative reductions in the UHR mode compared with the STD mode (4‐5% for Br44 and 8–9% for Br64).

**FIGURE 11:**
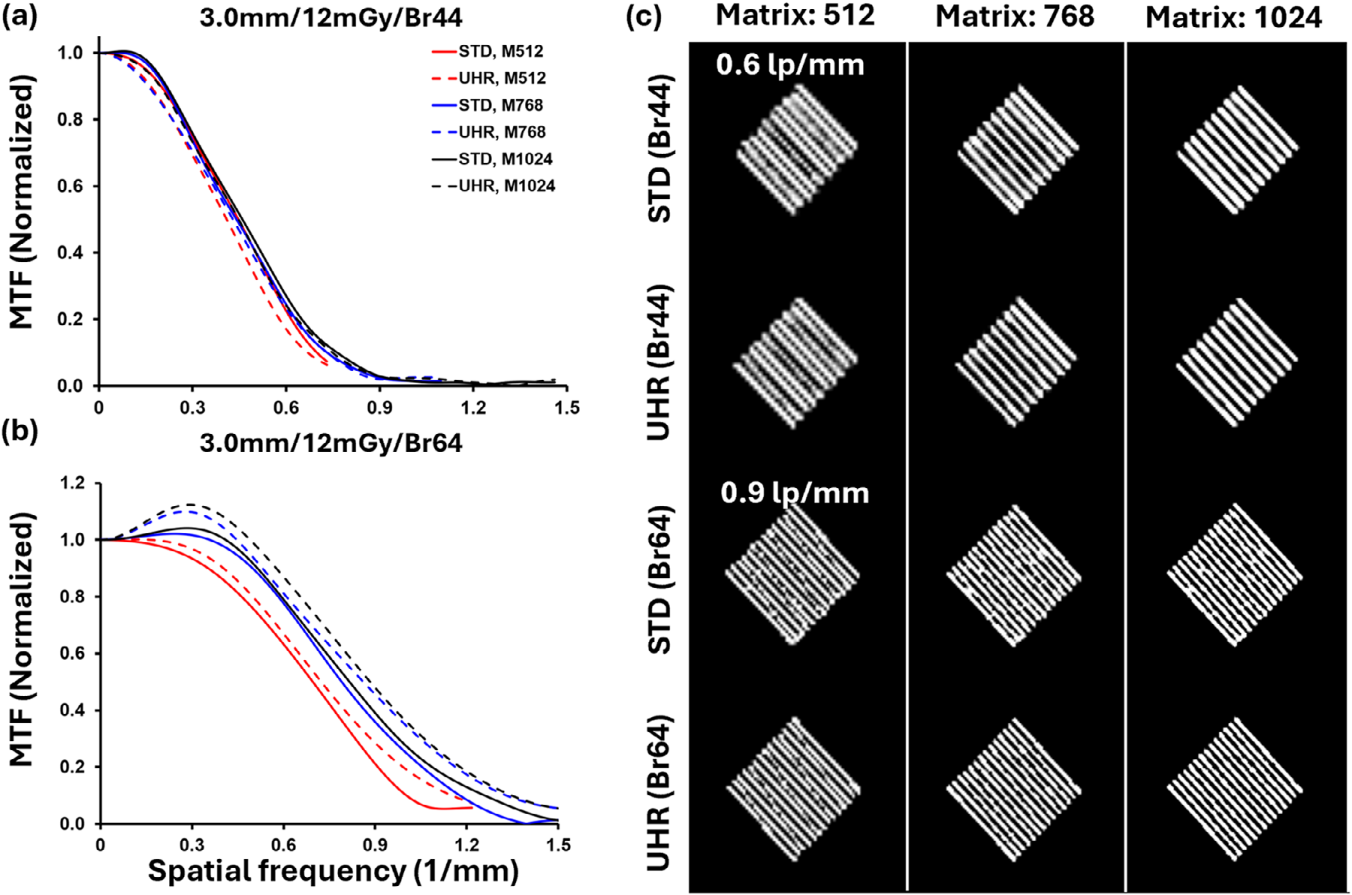
Representative MTF curves measured from T3D images and high‐contrast images for STD and UHR modes (3.0 mm slice thickness, 12 mGy) across matrix sizes (512 ‐ 1024). Results are shown for Br44 (a) and Br64 (b), with corresponding images of high‐contrast spatial resolution patterns (c).

MTF analysis demonstrated that increasing matrix size improved spatial resolution in both modes because of enhanced image sampling (Figure 11a,b; Table 4). For Br44, the UHR mode exhibited slightly lower spatial resolution than the STD mode, with MTF50 and MTF10 reduced by up to 7% and 5%, respectively. In contrast, for Br64, the UHR mode consistently improved spatial resolution across all matrix sizes, with MTF50 increasing by 4–7% and MTF10 by 9–16% relative to the corresponding STD mode. Representative high‐contrast spatial resolution patterns are shown in Figure 11c. Additional matrix‐dependent results across different imaging conditions are presented in Figures S12–S15.

### 3.6 Effect of virtual monoenergetic imaging

**FIGURE 12:**
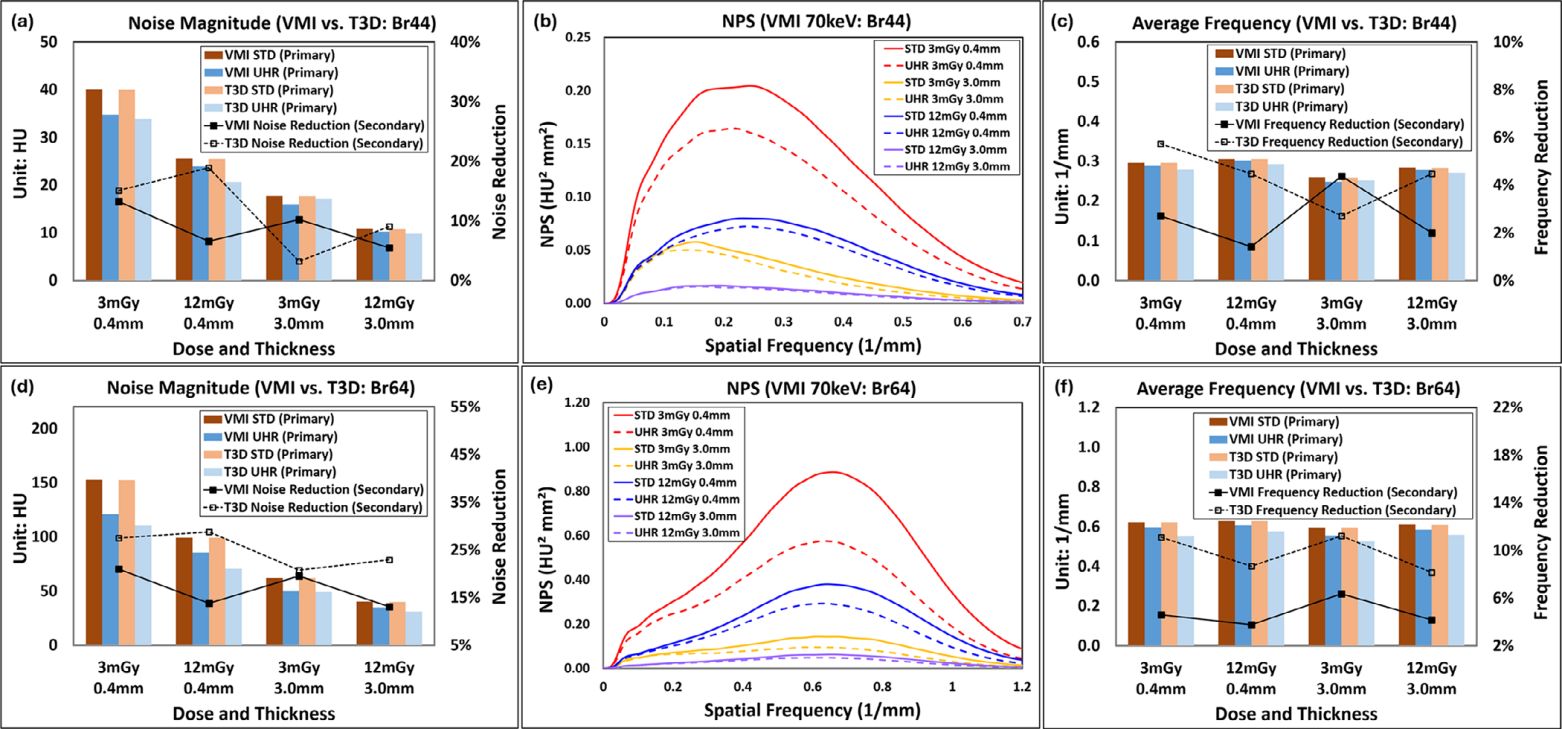
Comparison of VMI70 and T3D on noise magnitude, NPS, and average spatial frequency for Br44 and Br64 kernels. Results are shown for Br44 (a–c) and Br64 (d–f): noise magnitude (a, d), NPS (b, e), and average spatial frequency (c, f), with percentage reduction on the secondary axis. NPS curves in (b) and (e) correspond to VMI (70 keV) images. Supplementary Figure S16 presents the corresponding T3D and VMI (70 keV) NPS curves. Note: The standard deviation was negligible (SD < 0.3 HU for noise magnitude and SD < 0.002 mm^−^^1^ for average spatial frequency).

**FIGURE 13:**
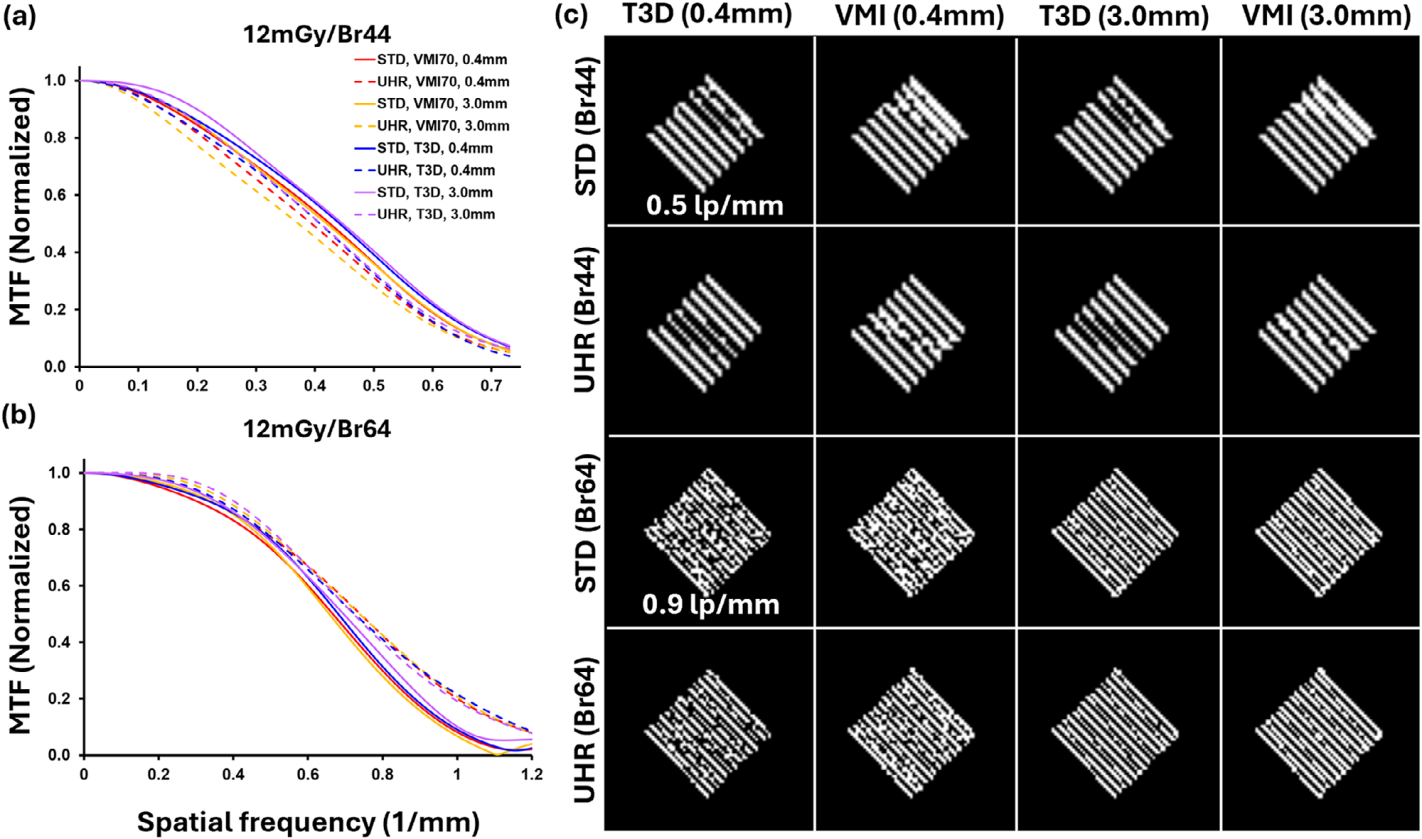
Representative MTF curves and high‐contrast images comparing VMI and T3D for Br44 (a) and Br64 (b) at 12 mGy across slice thicknesses, with corresponding images of high‐contrast spatial resolution patterns (c).

For both Br44 and Br64 kernels, image noise exhibited similar trends for VMI and T3D across all dose and slice thickness conditions (Figure 12a,d). Compared with the STD mode, the UHR mode consistently reduced image noise for VMI reconstructions, with reduction ranges comparable to those observed for T3D. Similarly, NPS analysis of VMI images demonstrated lower magnitude at thinner slice thickness while preserving the spectral shape (Figure 12b,e), consistent with slight reduction in average spatial frequency (Figure 12c,f). MTF analysis and high‐contrast spatial resolution patterns demonstrated spatial resolution characteristics comparable to those of T3D (Figure 13; Table 5), indicating that the observed effects of the UHR mode are also applicable to spectral reconstructions such as VMIs. Additional comparisons between VMI and T3D reconstructions are presented in Figure S16 and S17.

**TABLE 5:** MTF Metrics for STD and UHR Modes at VMI70 for Different Dose, Kernel, and Slice Thickness Settings.

		STD (1/mm)		UHR (1/mm)		Difference (%)	
		MTF50%	MTF10%	MTF50%	MTF10%	MTF50%	MTF10%
VMI70 (3mGy/Br44)	0.4 mm	0.337	0.607	0.296	0.566	−12.2	−6.7
	3.0 mm	0.337	0.620	0.295	0.568	−12.3	−8.5
VMI70 (12mGy/Br44)	0.4 mm	0.425	0.693	0.392	0.653	−7.8	−5.7
	3.0 mm	0.418	0.695	0.379	0.648	−9.2	−6.7
VMI70 (3mGy/Br64)	0.4 mm	0.542	0.896	0.501	0.972	−7.6	8.4
	3.0 mm	0.533	0.885	0.496	0.973	−6.8	9.9
VMI70 (12mGy/Br64)	0.4 mm	0.666	0.971	0.755	1.153	13.2	18.7
	3.0 mm	0.667	0.973	0.747	1.160	12.0	19.2

Values represent the mean of three repeated measurements.

Difference (%) = (UHR − STD) / STD × 100.

### 3.7 Effect of field of view

**FIGURE 14:**
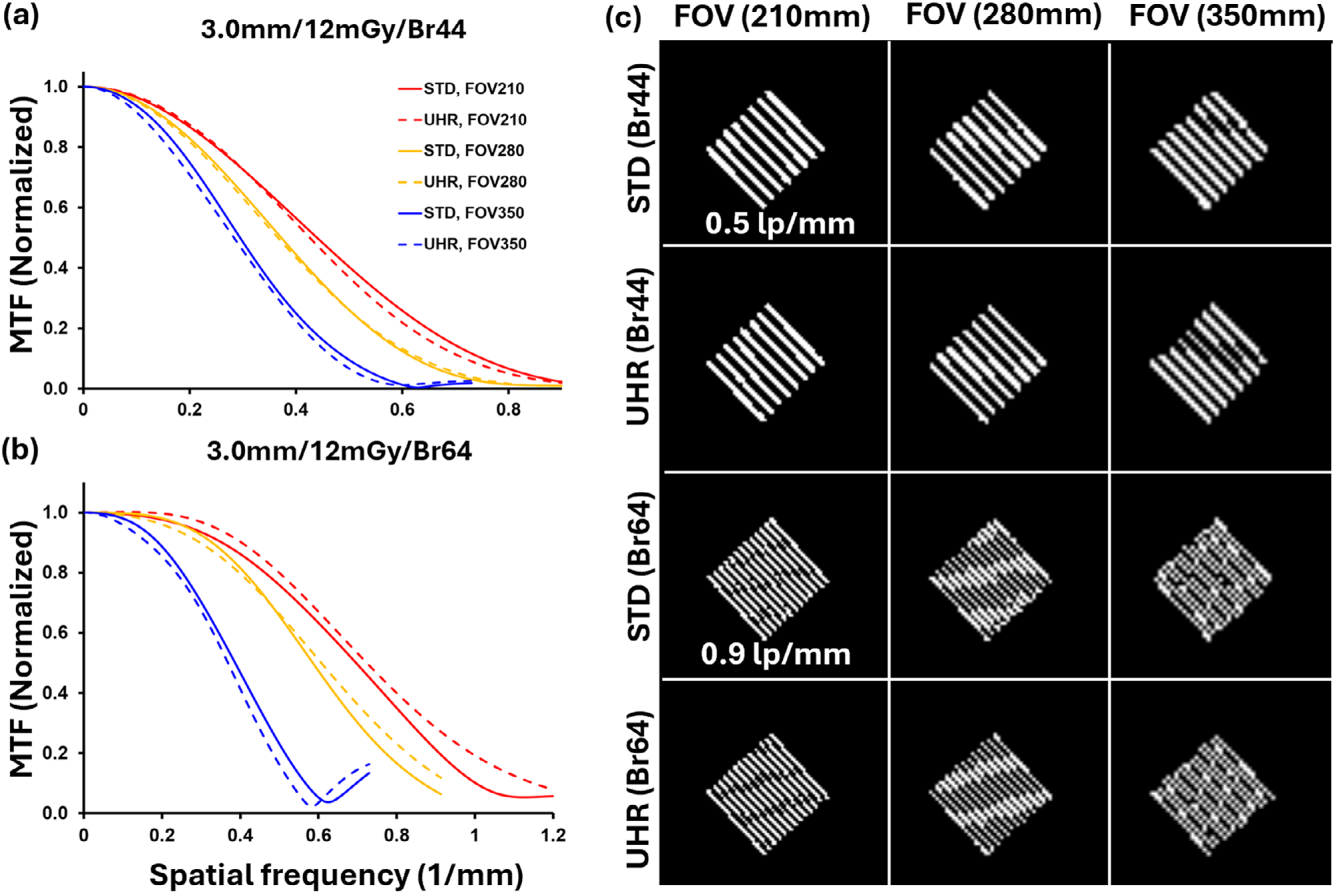
Representative MTF curves measured from T3D images and high‐contrast images for STD and UHR modes (3.0 mm slice thickness, 12 mGy) across FOVs (210–350 mm). Results are shown for Br44 (a) and Br64 (b), with corresponding images of high‐contrast spatial resolution patterns (c).

For both Br44 and Br64 kernels, increasing FOV resulted in progressive degradation of spatial resolution, as reflected by a leftward shift of the MTF curves and reductions in both MTF50 and MTF10 (Figure 14a,b). For Br44, both the STD and UHR modes showed a decrease of 34% in MTF10 from 210 to 350 mm. Compared with the STD mode, the UHR mode exhibited comparable spatial resolution across all FOVs, with MTF50 differences ranging from ‐1.6% to ‐3.9%. MTF10 was slightly higher at 210 mm (+4.4%) and 280 mm (+1.7%), but was 4.7% lower at 350 mm.

For Br64, spatial resolution likewise decreased with increasing FOV. MTF10 decreased by 42% for the STD mode and 53% for the UHR mode. Compared with the STD mode, the UHR mode demonstrated improved spatial resolution at smaller FOVs, with MTF50 increasing by 3.7‐4.3% and MTF10 by 11.7‐15.7% at 210 and 280 mm. However, at 350 mm, the UHR mode exhibited lower spatial resolution than the STD mode, with MTF50 and MTF10 reduced by 4.8% and 6.7%, respectively. Qualitative assessment of the high‐contrast spatial patterns (Figure 14c) was consistent with the quantitative findings. At smaller FOVs, high‐contrast patterns were well resolved in both modes, with sharper edges definition in the UHR mode, particularly for Br64. As FOV increased, fine structures became progressively blurred, with the greatest degradation observed at 350 mm. Additional FOV‐dependent results across multiple imaging conditions are presented in Figure S18.

## 4 DISCUSSION

This phantom study provides a systematic, multi‐parametric evaluation of the small‐pixel effect in a PCCT system, enabling direct assessment of how acquisition and reconstruction parameters influence image quality across a wide range of imaging conditions, including dose, slice thickness, reconstruction kernel, IR strength, matrix size, and FOV.

The present results demonstrated that the noise reduction associated with the UHR mode persisted across a broad range of imaging conditions, although its magnitude varied with acquisition and reconstruction parameters.^6^, ^10^ Across all imaging conditions, the UHR mode showed the greatest reductions observed for thin slice thickness and sharp reconstruction kernels. These findings are consistent with previous studies of lung, spine, and vascular PCCT imaging, which likewise reported lower noise and improved CNR with the UHR mode.^4^, ^8^, ^11^, ^14^ For example, Huflage et al. demonstrated substantial noise reduction and improved depiction of lung structures using UHR acquisitions,^4^ while Fix Martinez et al. reported dose reductions of up to 90% when UHR data were reconstructed to matched spatial resolution.^8^ NPS analysis demonstrated that the UHR mode reduced overall noise magnitude and, depending on imaging conditions, shifted the noise spectrum toward lower spatial frequencies.^6^, ^22^ This shift toward lower average spatial frequency indicates relative suppression of high‐frequency noise, resulting in a smoother image appearance that may influence the perceived sharpness of anatomical structures. Therefore, evaluation of spatial resolution is essential.

Spatial resolution, quantified by MTF50 and MTF10, showed only limited differences between the STD and UHR modes across slice thicknesses up to 3.0 mm. Increasing matrix size had minimal impact on noise characteristics at a fixed FOV but improved spatial resolution through enhanced image sampling. The small differences between the STD and UHR modes suggest that the intrinsic detector‐level resolution advantage of smaller pixels does not consistently translate into higher measured MTF under matched reconstruction settings, consistent with previous reports that UHR PCCT primarily preserves nominal system resolution while reducing image noise.^12^, ^23^ These results emphasize that reconstruction FOV and matrix size should be selected to achieve sufficiently small image pixel sizes when sharp reconstruction kernels are used. Otherwise, high‐spatial‐frequency information may be inadequately sampled, limiting the potential benefit of UHR imaging despite the intrinsic detector resolution. The minimal differences observed between VMI and T3D reconstructions suggest that reconstruction mode has a greater influence on image quality than spectral reconstruction approach under the evaluated conditions. This indicates that detector sampling and reconstruction parameters primarily determine noise and spatial resolution, while spectral processing has a comparatively smaller contribution.^24^

The observed differences between the STD and UHR modes have important implications for clinical protocol optimization. The UHR mode demonstrated the greatest noise reduction at thinner slice thicknesses and with sharper reconstruction kernels, where higher spatial sampling is maintained. These acquisition settings are commonly used for high‐resolution imaging tasks, including evaluation of the lung parenchyma, small airways, interstitial lung disease, fine vascular anatomy, temporal bone, and musculoskeletal structures, where improved noise performance may enhance the visualization and detectability of subtle anatomical details.^2^, ^8^, ^11^ Therefore, the choice between the STD and UHR modes should be guided by the specific clinical task, balancing the need for spatial resolution, image noise, and workflow efficiency while considering factors such as slice thickness, reconstruction kernel, radiation dose, and image sampling.^1^, ^25^ In addition, differences in detector collimation between the STD and UHR modes may further influence dose efficiency and image quality and should be considered during protocol optimization.

This study has several limitations. First, although the phantom‐based design provides excellent standardization and reproducibility, it does not fully capture anatomical variability, heterogeneous attenuation, or other patient‐specific factors encountered in clinical imaging. Future studies incorporating phantoms of different sizes, anthropomorphic phantoms, or patient datasets would provide a more comprehensive evaluation of UHR performance under clinically relevant conditions. Second, spectral reconstruction was evaluated only using 70 keV VMIs, representing a single spectral reconstruction. A comprehensive assessment of spectral imaging performance, including VMIs across multiple energy levels, iodine maps, virtual non‐contrast images, and other advanced spectral reconstructions, was beyond the scope of this study and warrants future investigation.

## 5 CONCLUSION

This study demonstrates that the UHR mode in PCCT enhances noise suppression across a broad range of imaging conditions, with the greatest reductions observed at thinner slice thicknesses and with sharper reconstruction kernels. These findings suggest that the UHR mode is best suited for high‐spatial‐resolution imaging tasks requiring thin slice thicknesses and sharp reconstruction kernels, where improved noise performance can be leveraged to maximize image quality. In contrast, the STD mode remains a more practical choice for routine CT examinations, where the additional noise reduction provided by the UHR mode is modest and is unlikely to provide meaningful clinical benefit.

## AUTHOR CONTRIBUTIONS

**Kyu‐Ho Song**: Conceptualization/study design; data acquisition; analysis and curation; writing—original draft; review and editing. **Colin Shan**: Conceptualization/study design; data acquisition; analysis and curation; writing—review and editing. **Gary Xu**: Data acquisition; analysis and curation; writing—review and editing. **Xinhui Duan**: Writing—review and editing. **Yue Zhang**: Data acquisition; analysis and curation; writing—review and editing. **Kuan Zhang**: Writing—review and editing. **Liqiang Ren**: Conceptualization/study design; data acquisition; analysis and curation; writing—original draft; review and editing.

## CONFLICT OF INTEREST STATEMENT

The authors declare no conflicts of interest.

## ETHICAL APPROVAL STATEMENT

This study did not involve human participants or animals and therefore did not require Institutional Review Board approval.

## Supporting information



Supporting Information

## Data Availability

The data supporting the findings of this study are available from the corresponding author upon reasonable request.
