# Supplementary material for: Phantom evaluation of small‐pixel effect in ultra‐high‐resolution photon‐counting CT: Noise texture and high‐contrast spatial resolution
Source: J Appl Clin Med Phys. 2026 Aug 28;27(9):e70776. doi: 10.1002/acm2.70776 (PMC13525204; doi:10.1002/acm2.70776)
Supplement: Supplementary file 1 [file ACM2-27-e70776-s001.pdf]

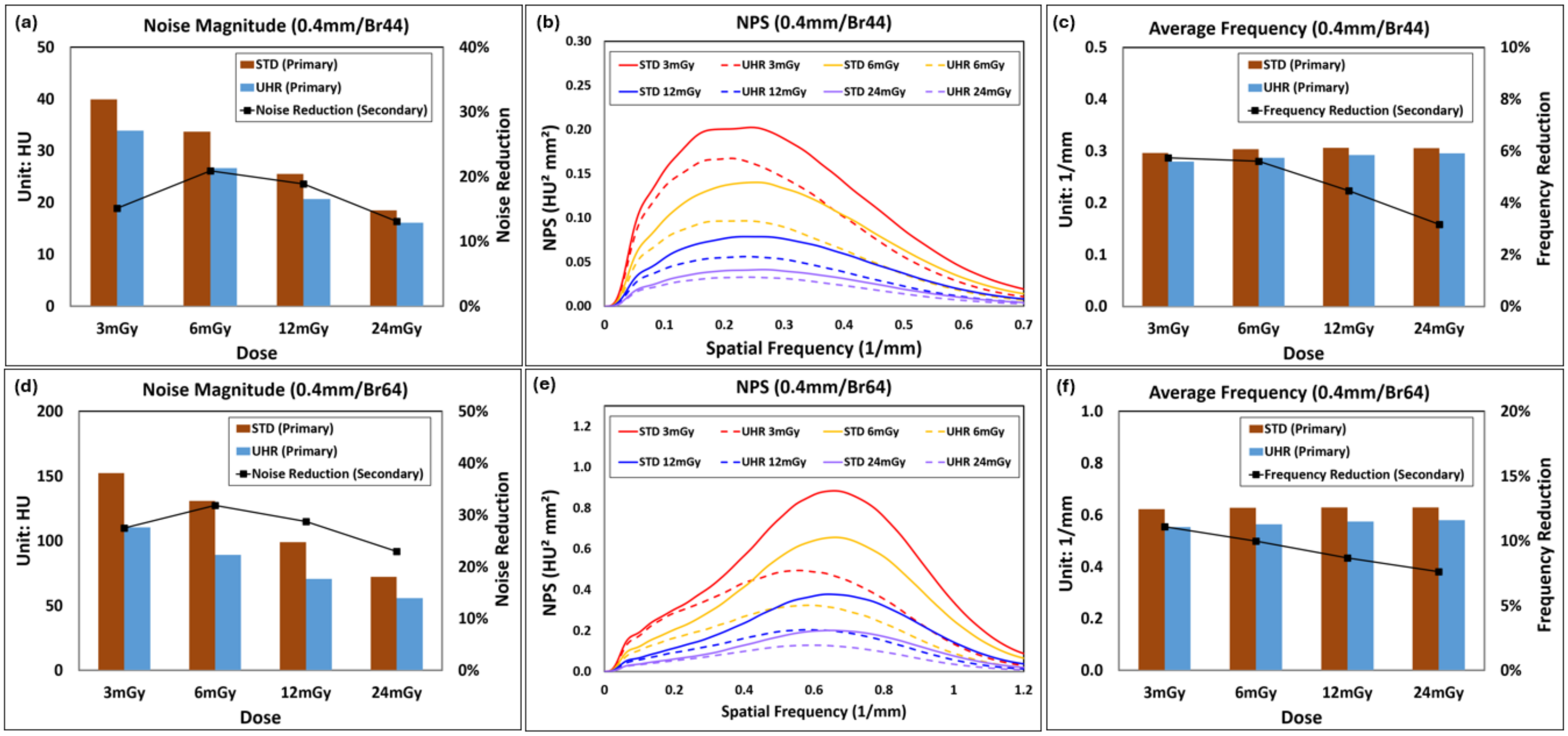

Figure S1. Effect of radiation dose on noise magnitude, NPS, and averaged spatial frequency at 0.4 mm slice thickness for Br44 (a–c) and Br64 (d–f) kernels. Secondary axes indicate relative noise and frequency reduction.

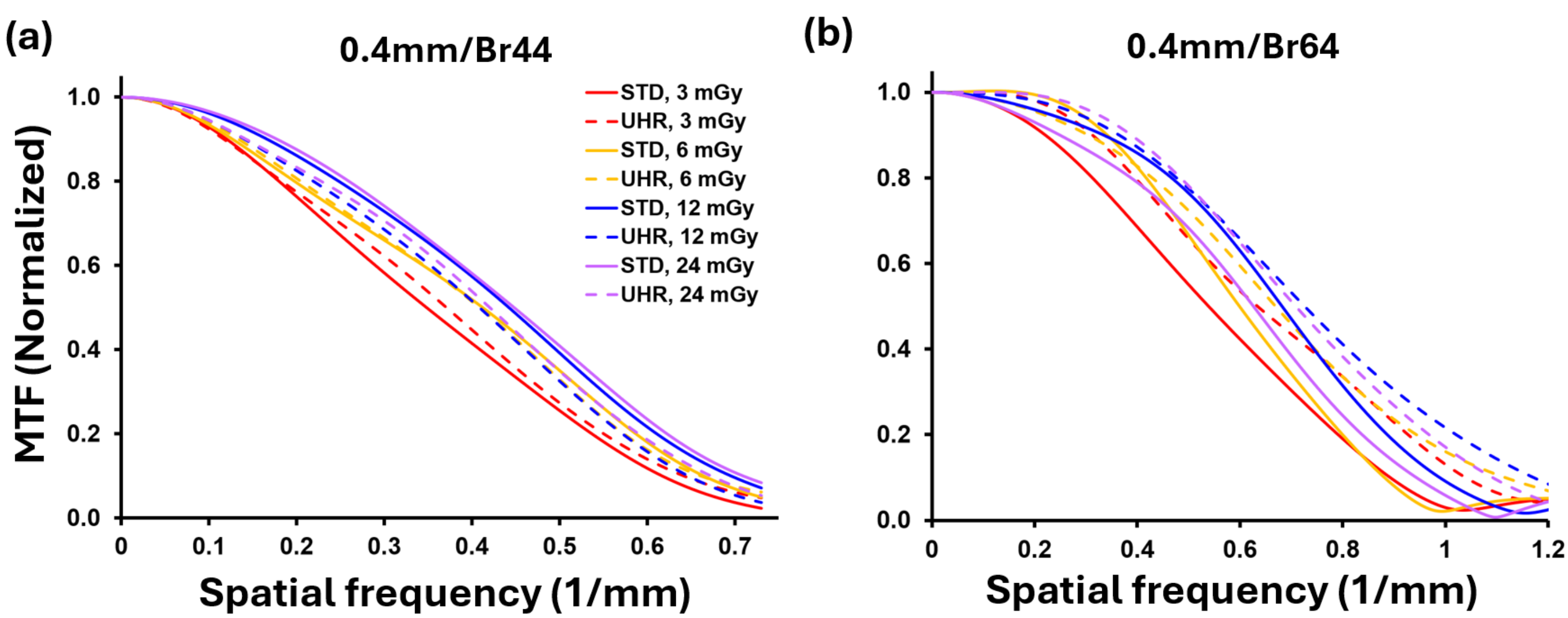

Figure S2. Effect of radiation dose on MTF at 0.4 mm slice thickness for Br44 (a) and Br64 (b) kernels. MTF curves for STD and UHR modes are shown across dose levels from 3 to 24 mGy.

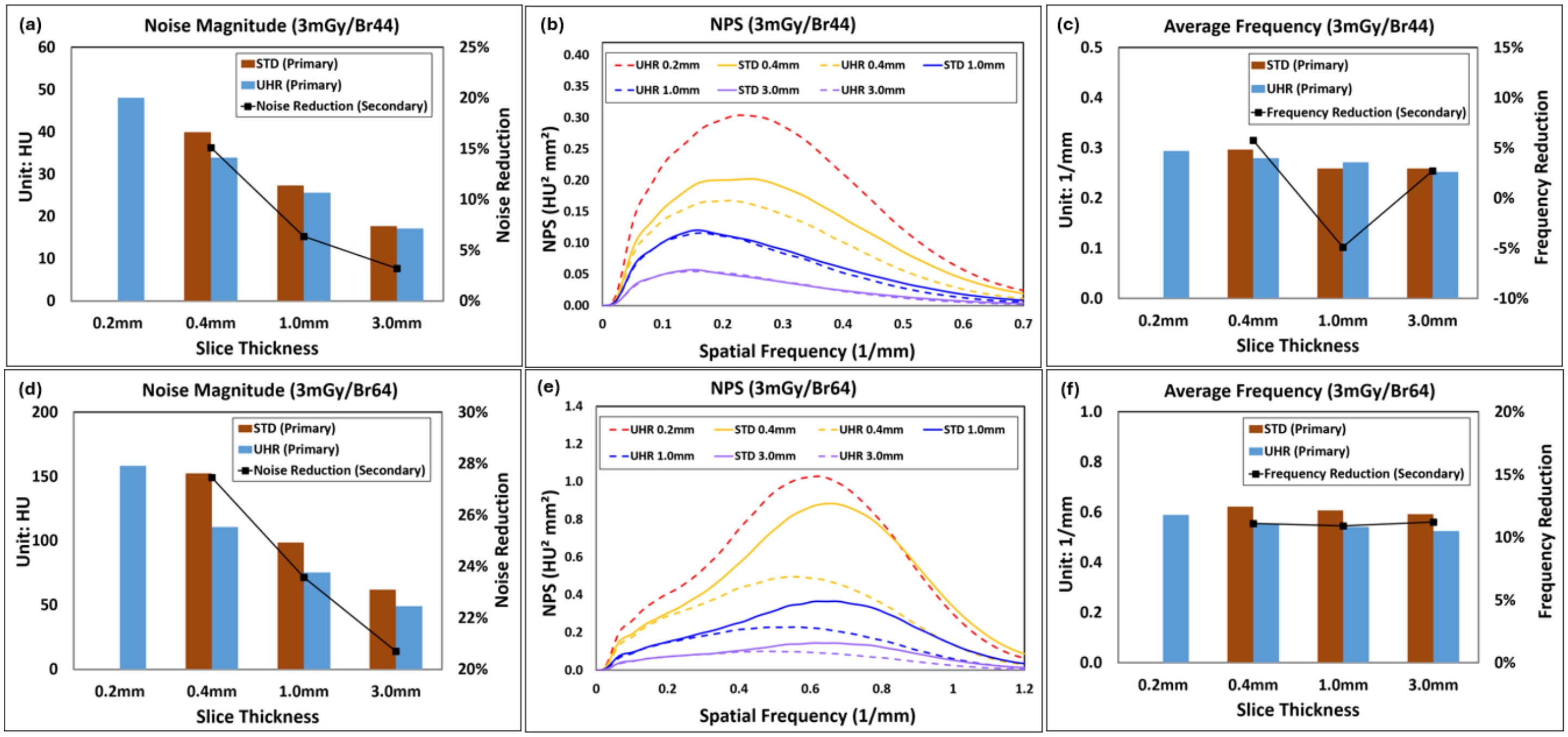

Figure S3. Effect of slice thickness on noise magnitude, NPS, and averaged spatial frequency at 3 mGy for Br44 (a–c) and Br64 (d–f) kernels. Secondary axes indicate relative noise and frequency reduction.

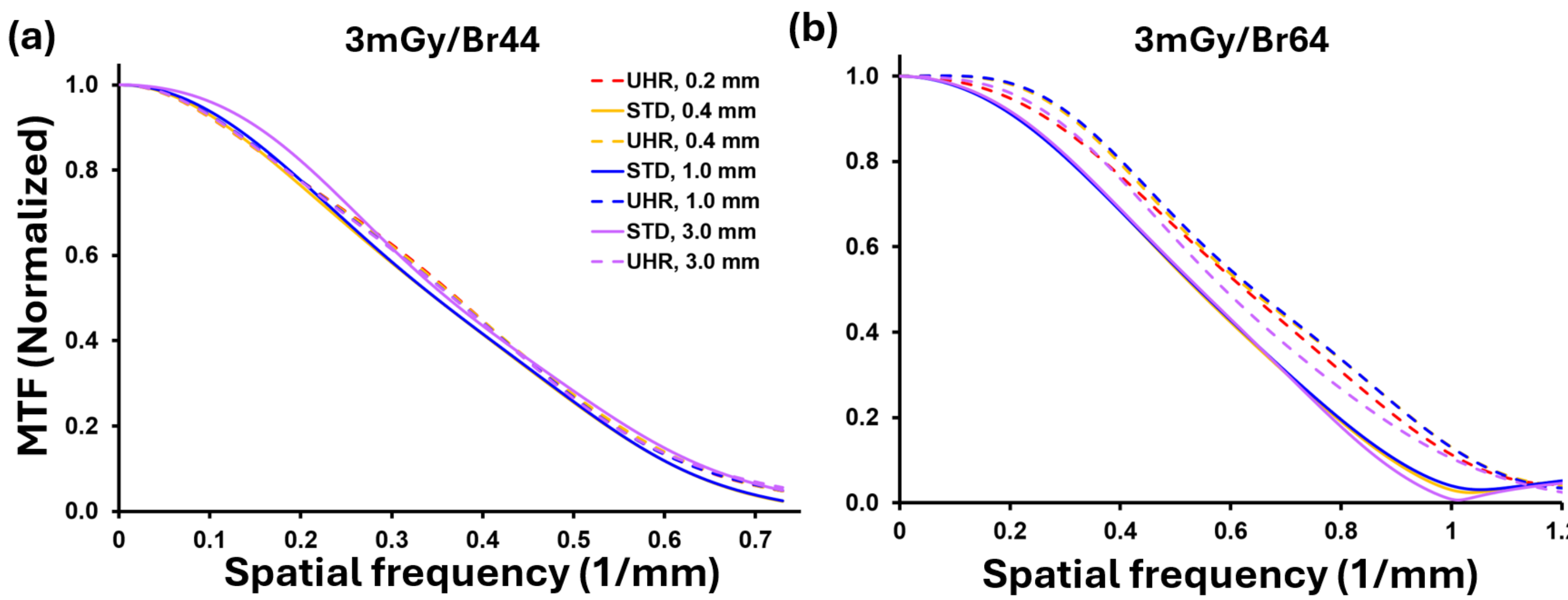

Figure S4. Effect of slice thickness on MTF at 3 mGy for Br44 (a) and Br64 (b) kernels. MTF curves for STD and UHR modes are shown across slice thicknesses from 0.2 to 3.0 mm.

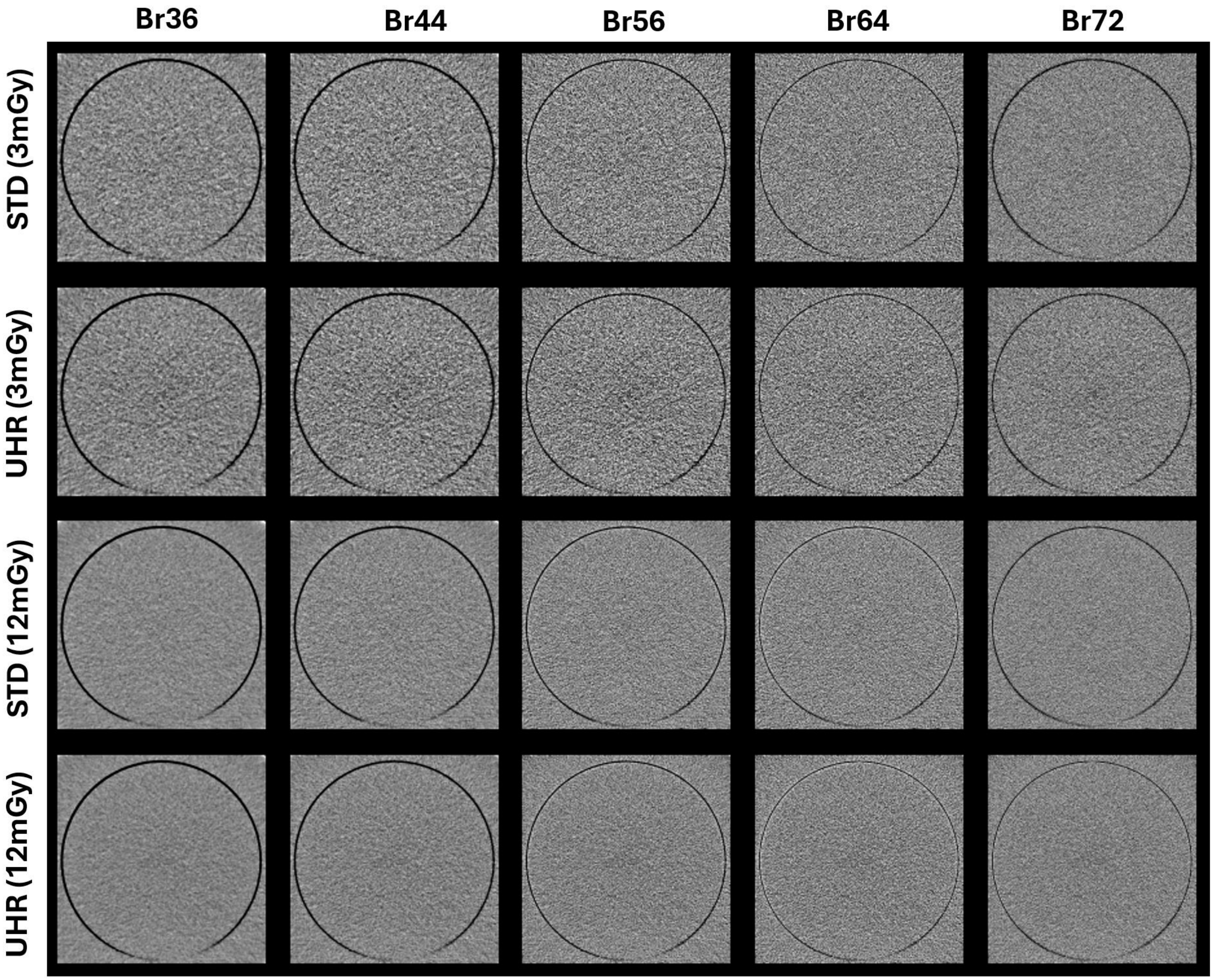

Figure S5. Representative noise texture images for STD and UHR modes across reconstruction kernels (Br36–Br72) at 3 mGy and 12 mGy. Rows correspond to STD (3 mGy), UHR (3 mGy), STD (12 mGy), and UHR (12 mGy), respectively. UHR images demonstrate reduced noise magnitude and smoother texture compared to STD, particularly for sharper kernels and lower dose conditions.

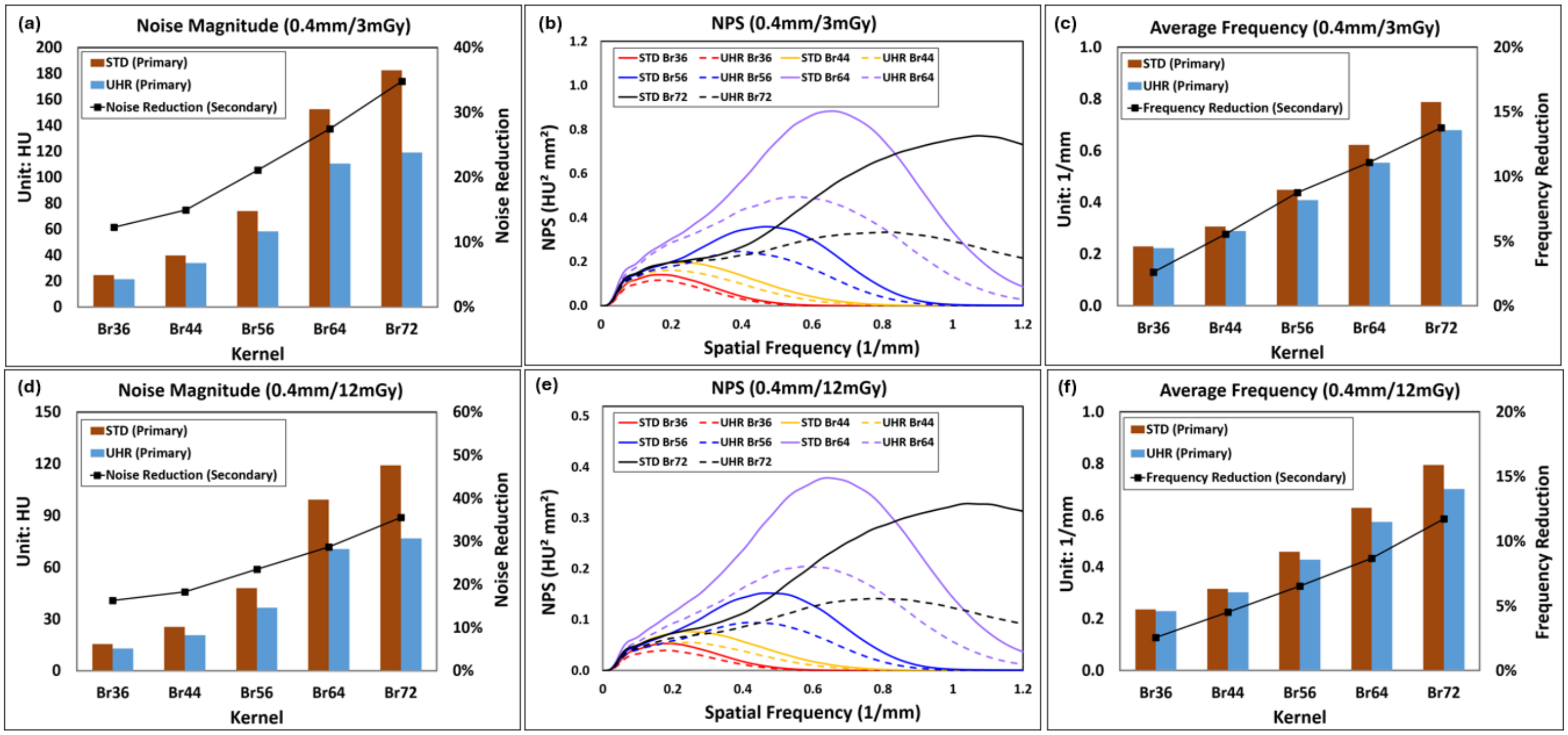

Figure S6. Effect of reconstruction kernel on noise magnitude, NPS, and averaged spatial frequency at 0.4 mm slice thickness for 3 mGy (a–c) and 12 mGy (d–f). Secondary axes indicate relative noise and frequency reduction.

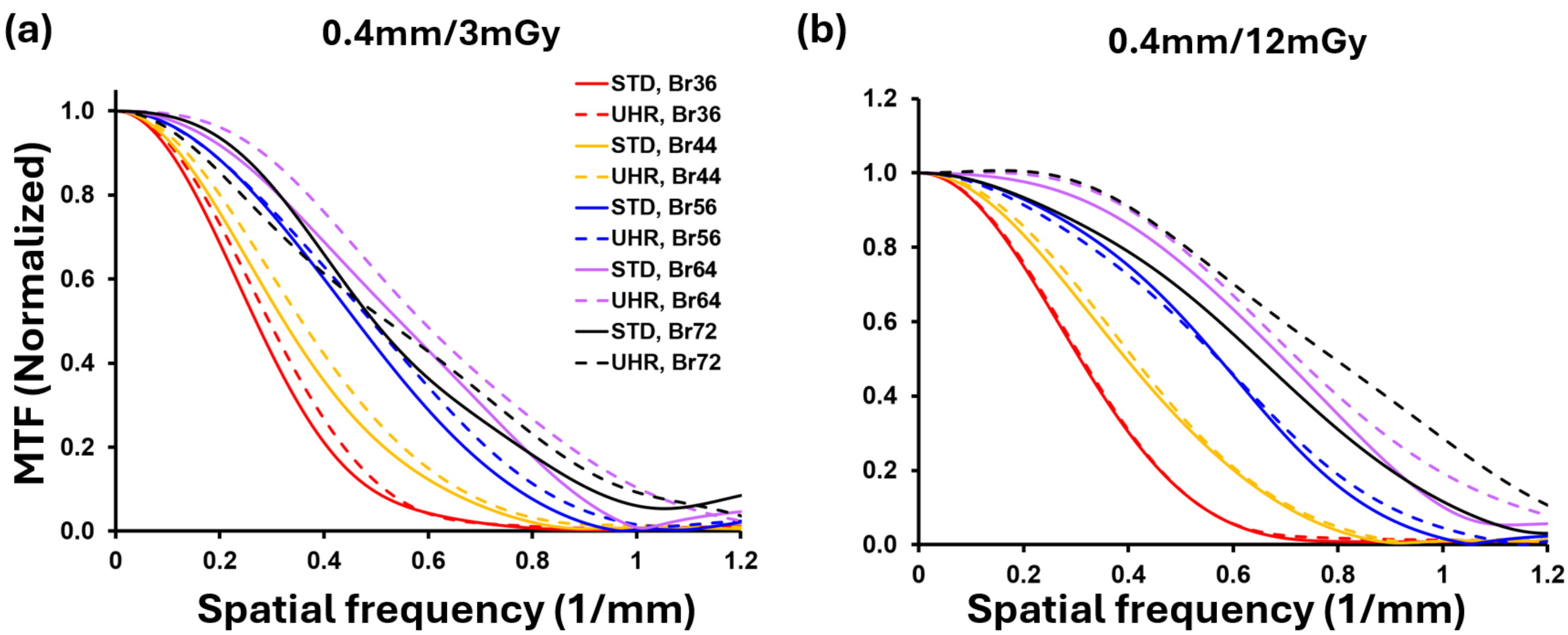

Figure S7. Effect of reconstruction kernel on MTF at 0.4 mm slice thickness for (a) 3 mGy and (b) 12 mGy. MTF curves for STD and UHR modes are shown across kernels from Br36 to Br72.

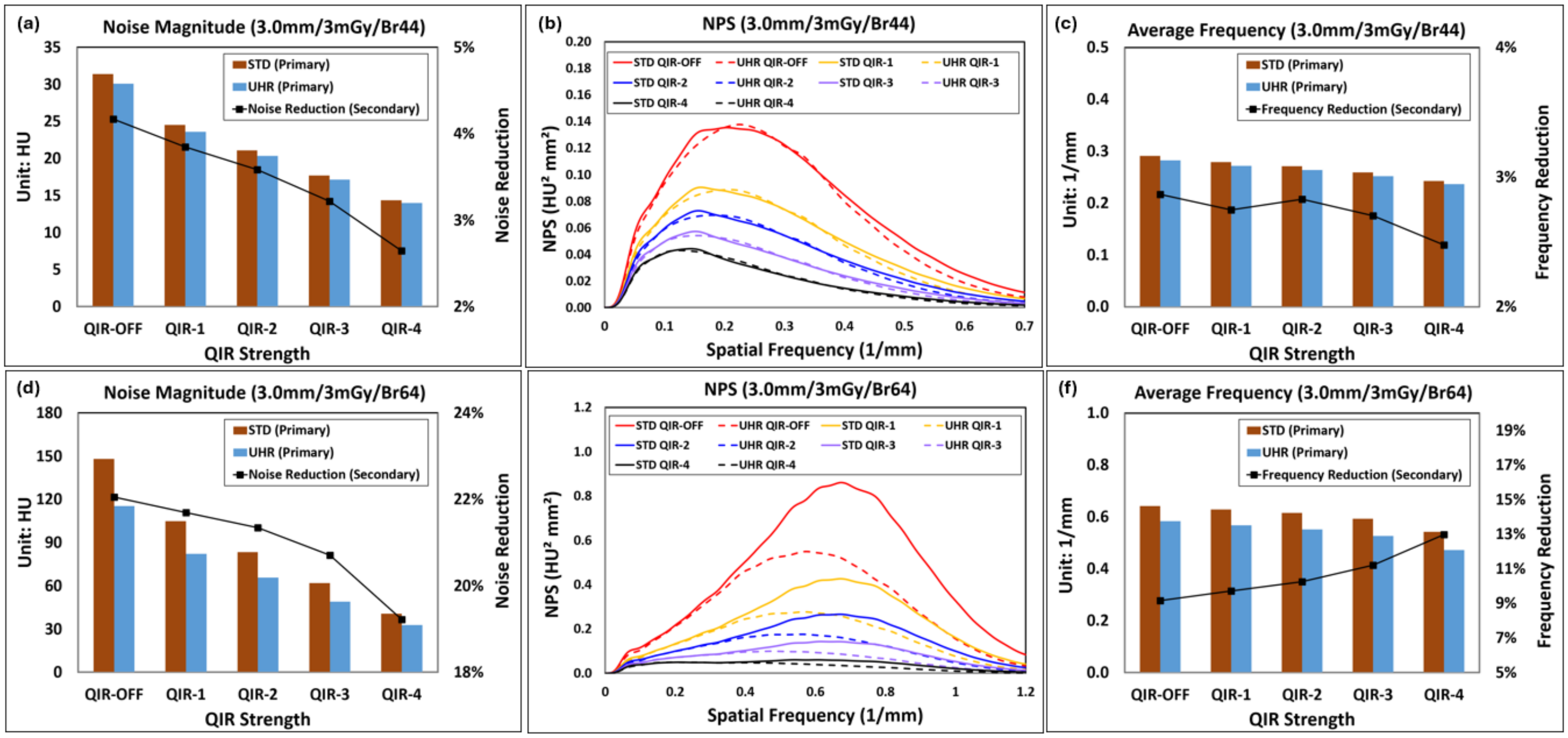

Figure S8. Effect of QIR strength on noise magnitude, NPS, and averaged spatial frequency at 3.0 mm and 3 mGy for Br44 (a–c) and Br64 (d–f). Secondary axes indicate relative noise and frequency reduction.

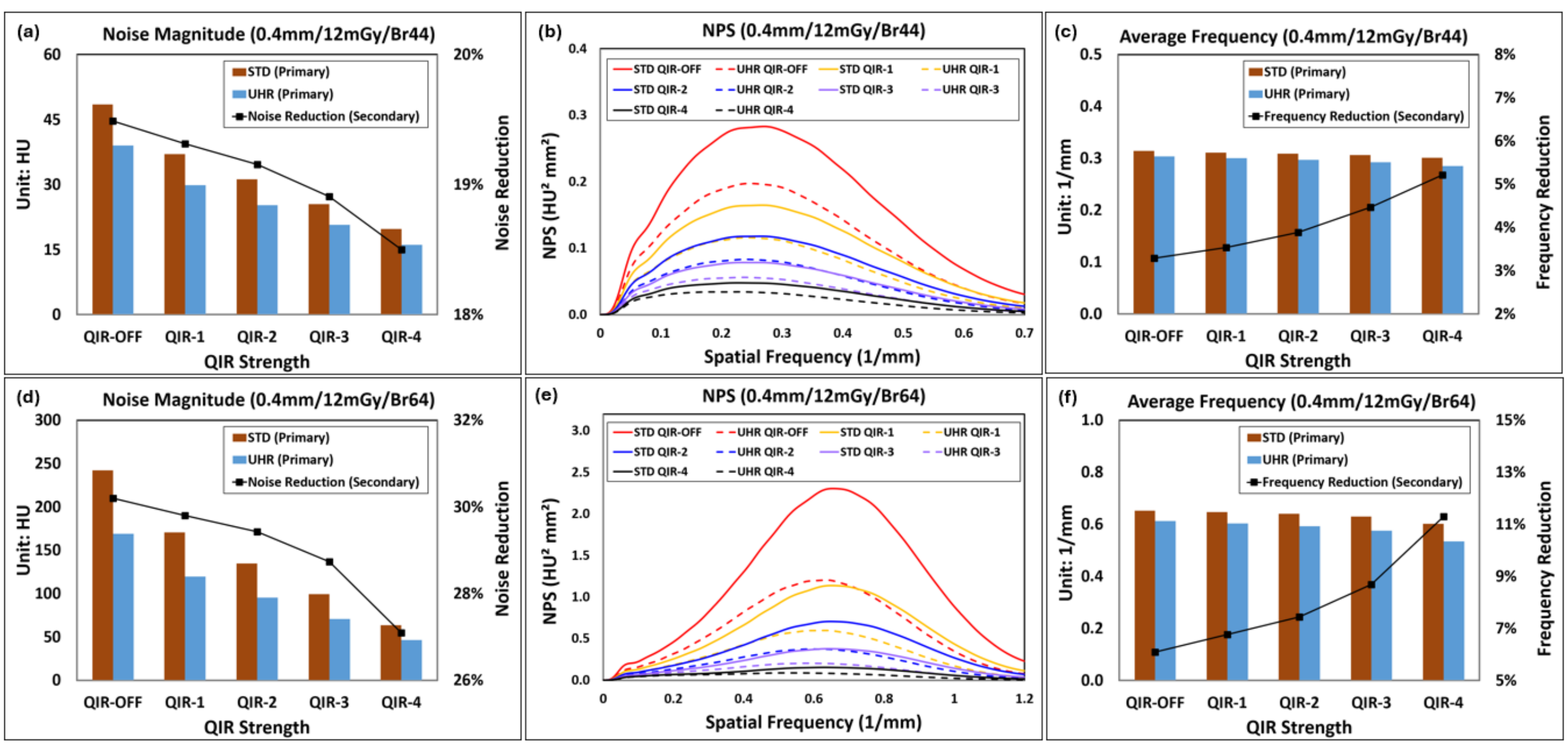

Figure S9. Effect of QIR strength on noise magnitude, NPS, and averaged spatial frequency at 0.4 mm and 12 mGy for Br44 (a–c) and Br64 (d–f). Secondary axes indicate relative noise and frequency reduction.

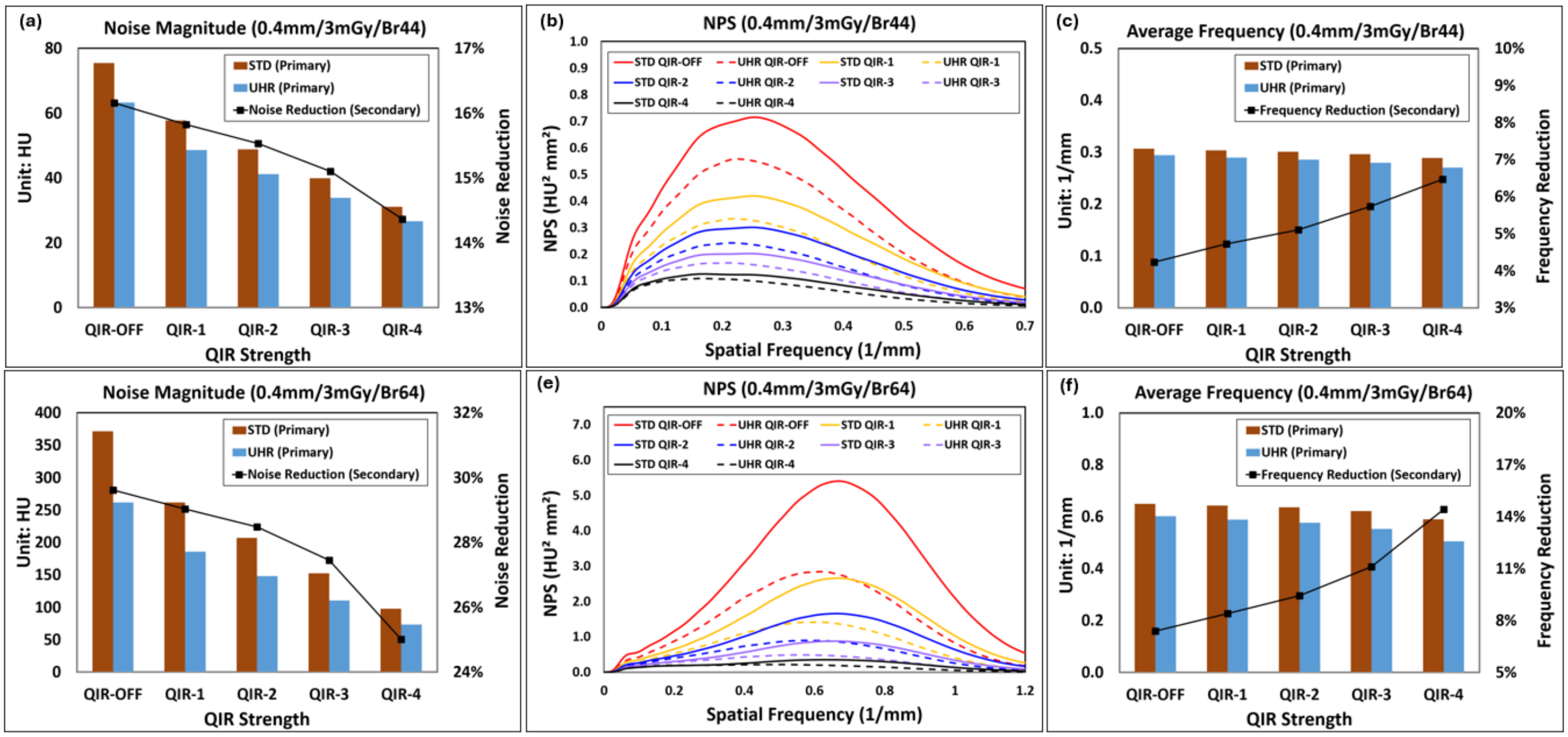

Figure S10. Effect of QIR strength on noise magnitude, NPS, and averaged spatial frequency at 0.4 mm and 3 mGy for Br44 (a–c) and Br64 (d–f). Secondary axes indicate relative noise and frequency reduction.

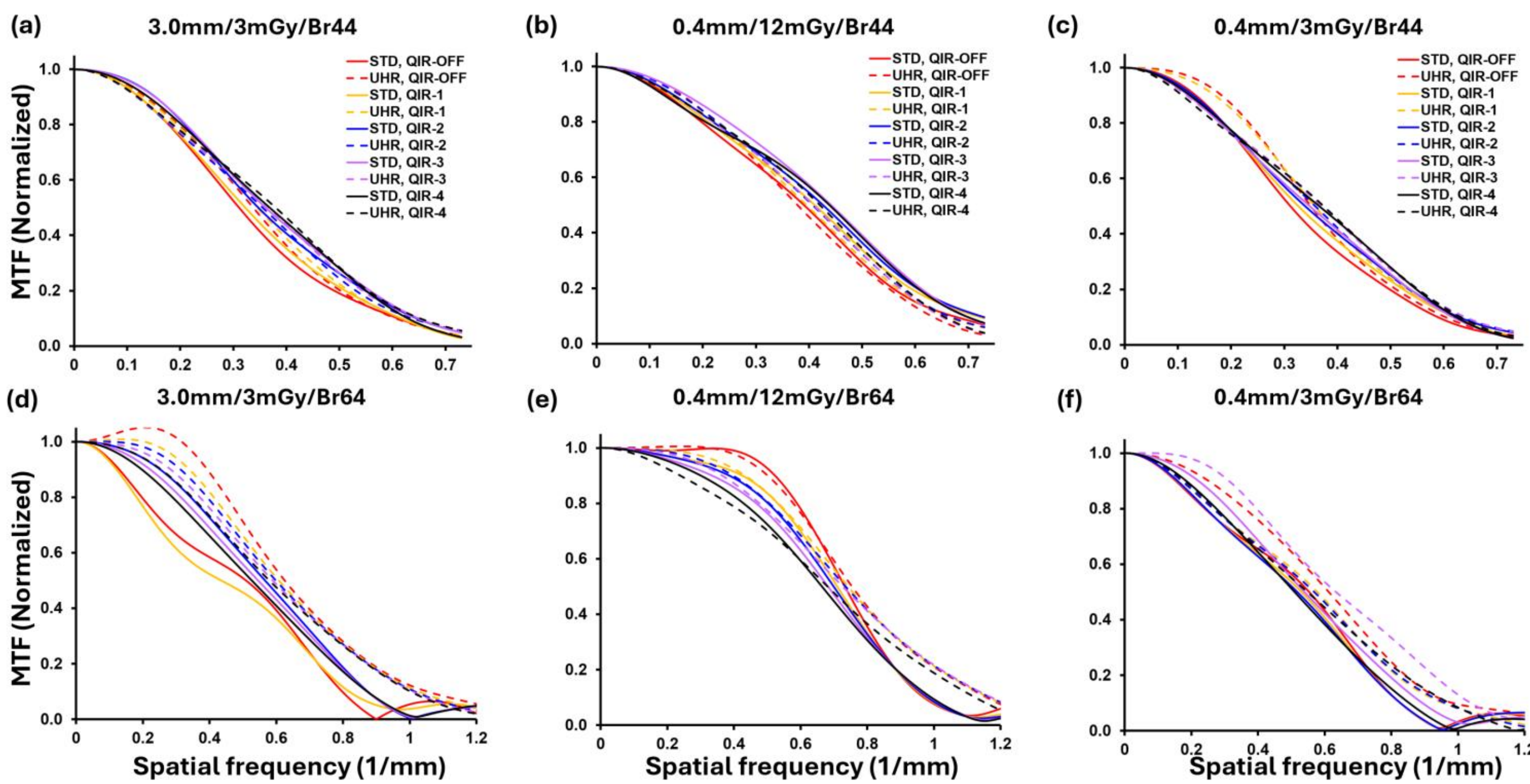

Figure S11. Effect of QIR strength on MTF for STD and UHR modes across multiple imaging conditions. Results are shown for Br44 (a–c) and Br64 (d–f) at different combinations of slice thickness and dose.

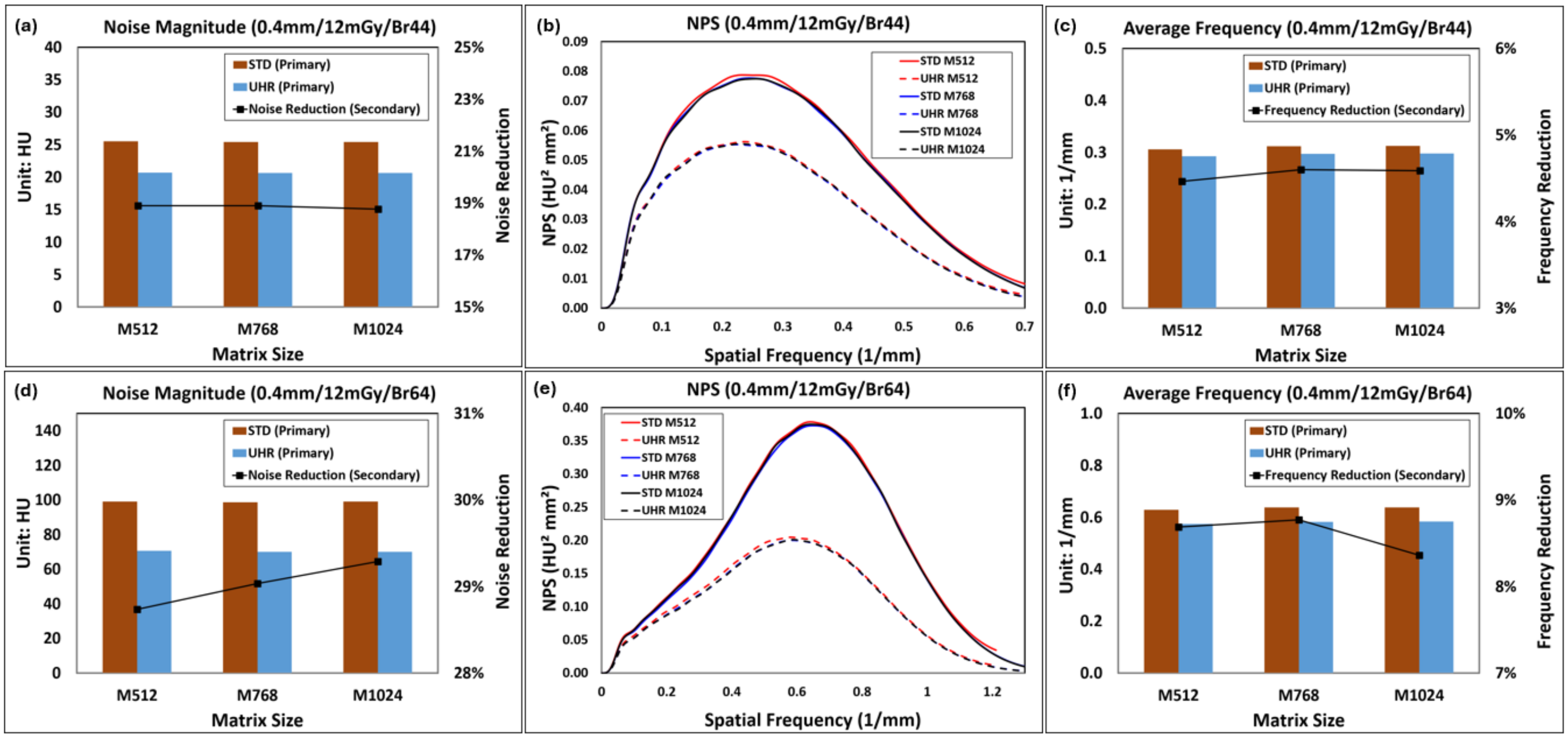

Figure S12. Effect of matrix size on noise magnitude, NPS, and averaged spatial frequency at 0.4 mm and 12 mGy for Br44 (a–c) and Br64 (d–f). Secondary axes indicate relative noise and frequency reduction.

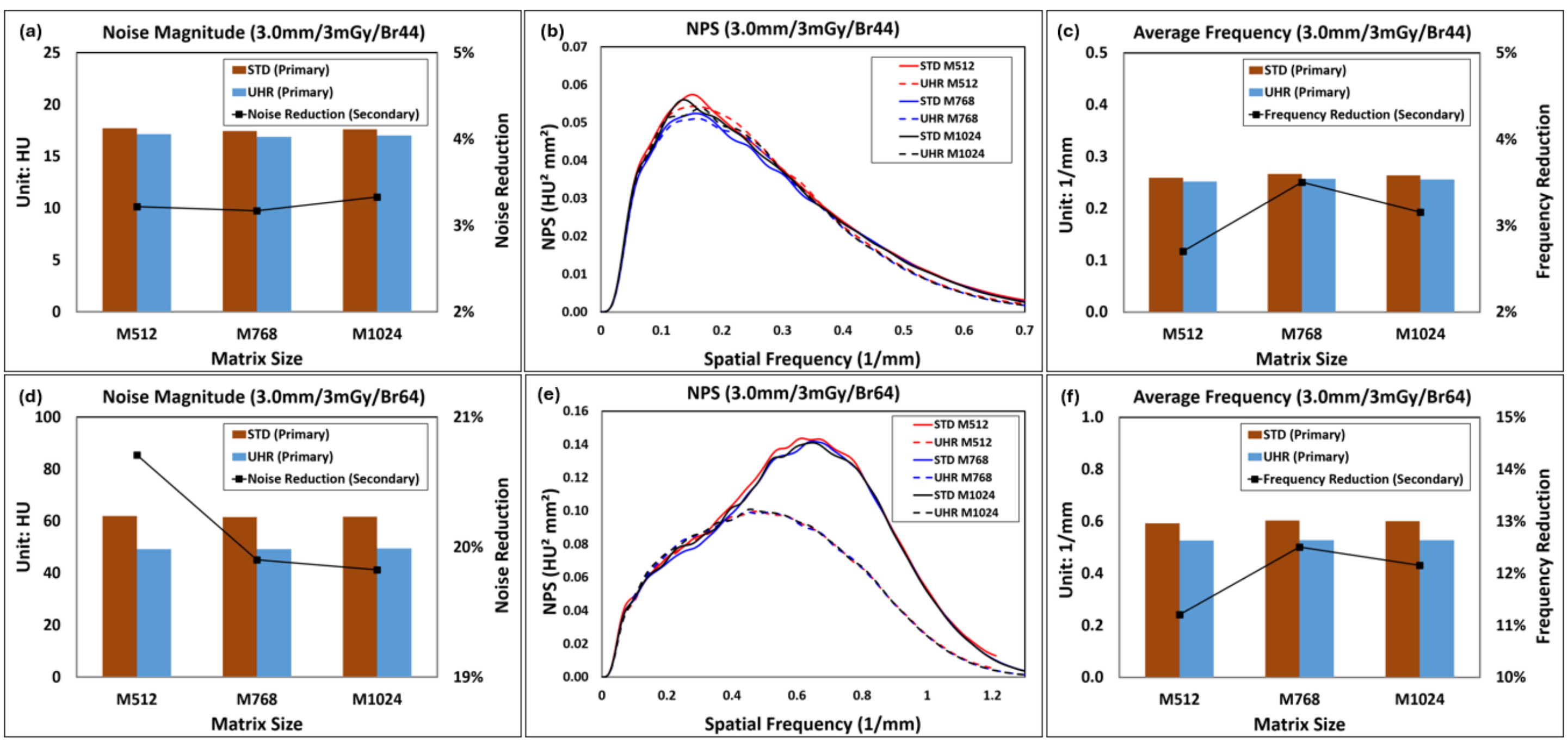

Figure S13. Effect of matrix size on noise magnitude, NPS, and averaged spatial frequency at 3.0 mm and 3 mGy for Br44 (a–c) and Br64 (d–f). Secondary axes indicate relative noise and frequency reduction.

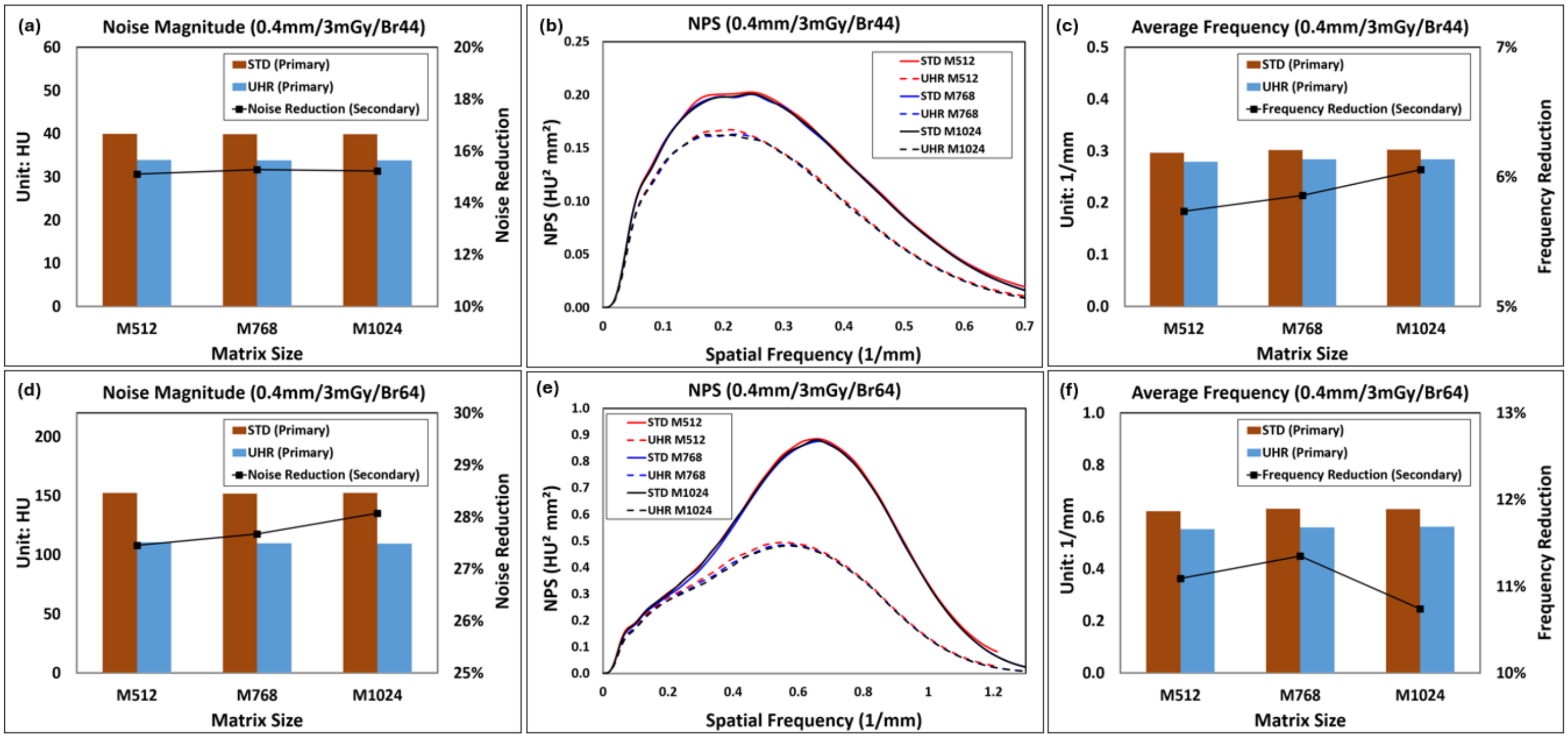

Figure S14. Effect of matrix size on noise magnitude, NPS, and averaged spatial frequency at 0.4 mm and 3 mGy for Br44 (a–c) and Br64 (d–f). Secondary axes indicate relative noise and frequency reduction.

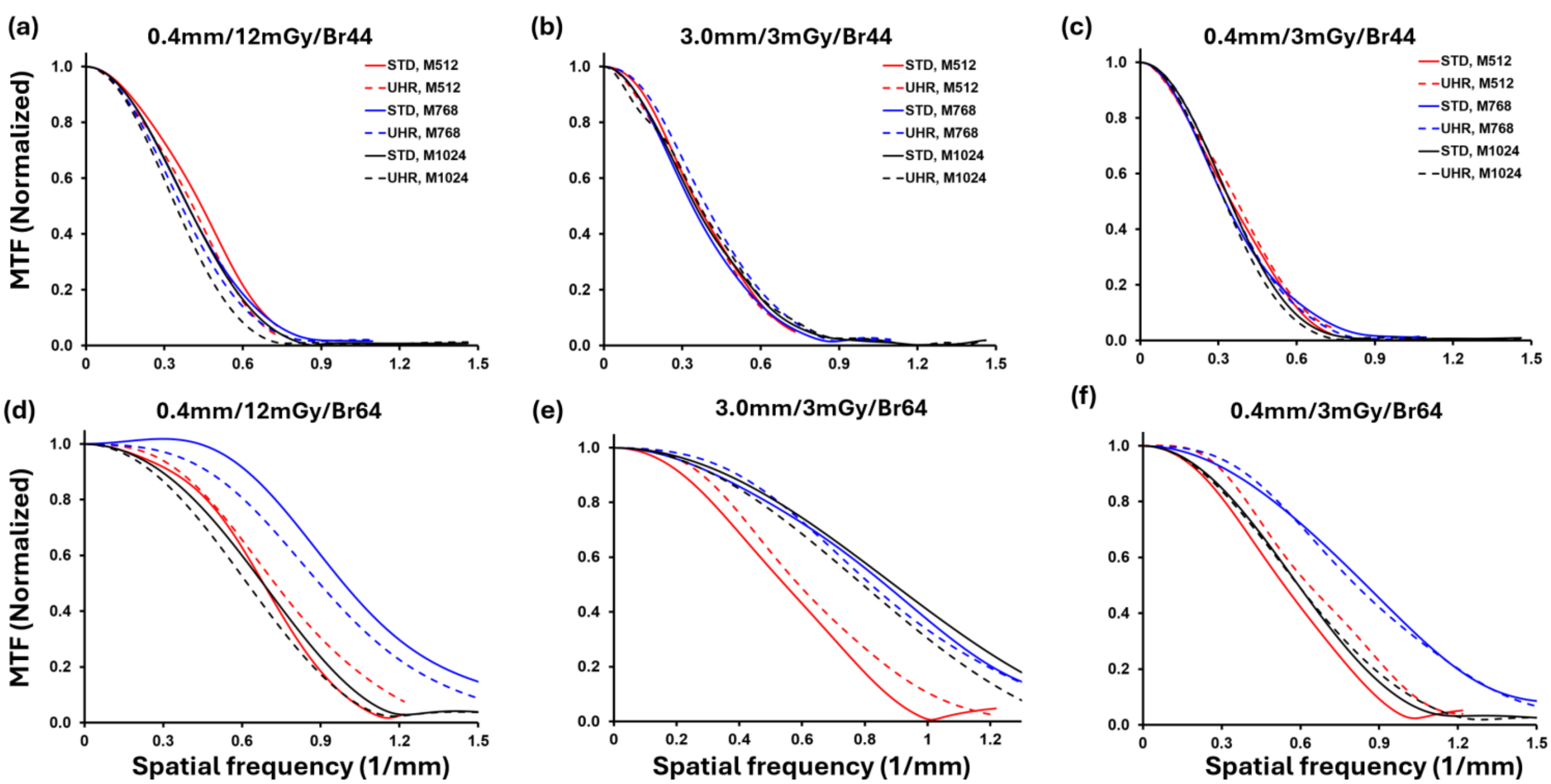

Figure S15. Effect of matrix size on MTF for STD and UHR modes across multiple imaging conditions. Results are shown for Br44 (a–c) and Br64 (d–f) at different combinations of slice thickness and dose.

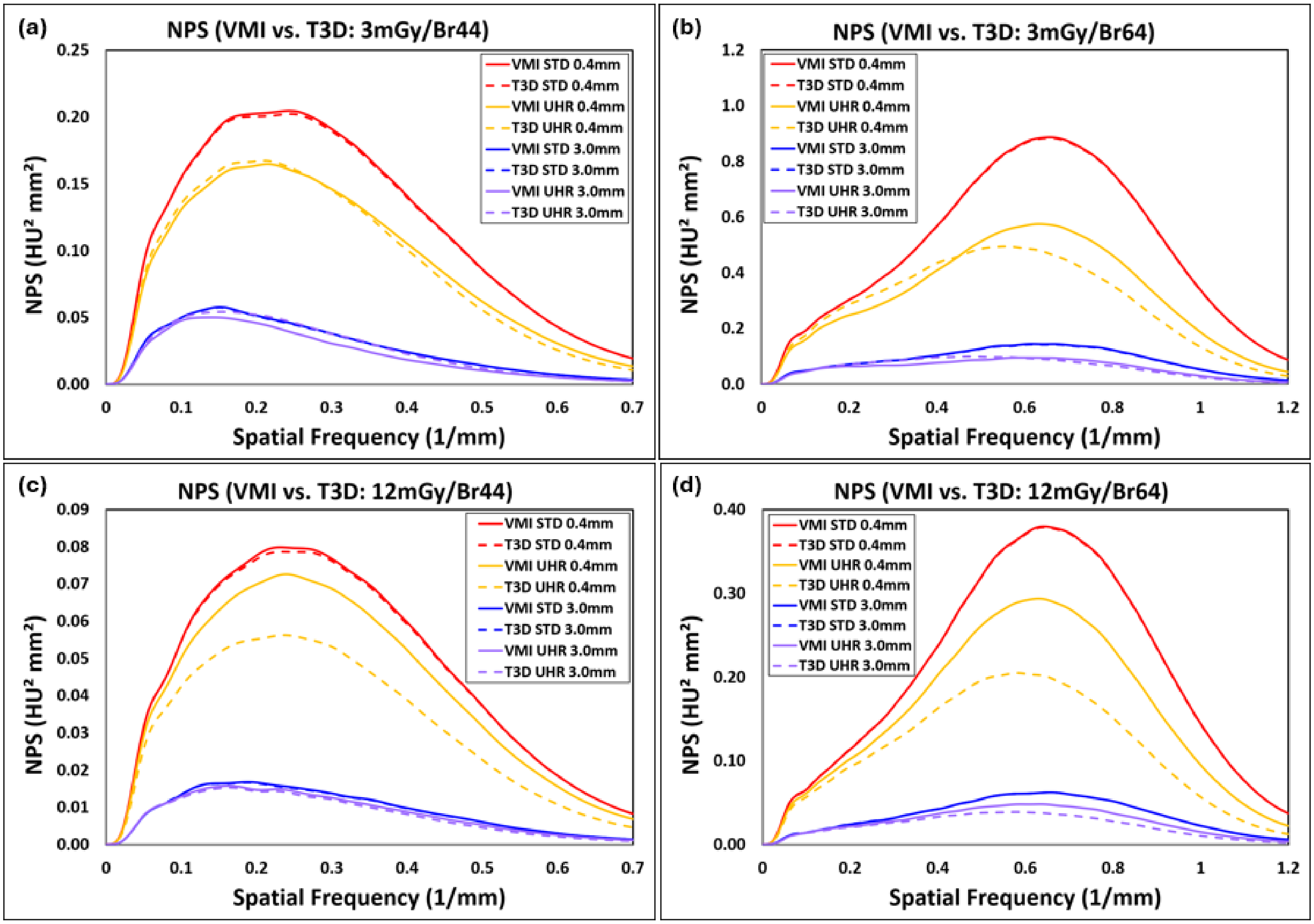

Figure S16. NPS comparison between VMI and T3D reconstructions for (a) Br44 at 3 mGy, (b) Br64 at 3 mGy, (c) Br44 at 12 mGy, and (d) Br64 at 12 mGy. Results are shown for both STD and UHR acquisition modes at 0.4 and 3.0 mm slice thickness.

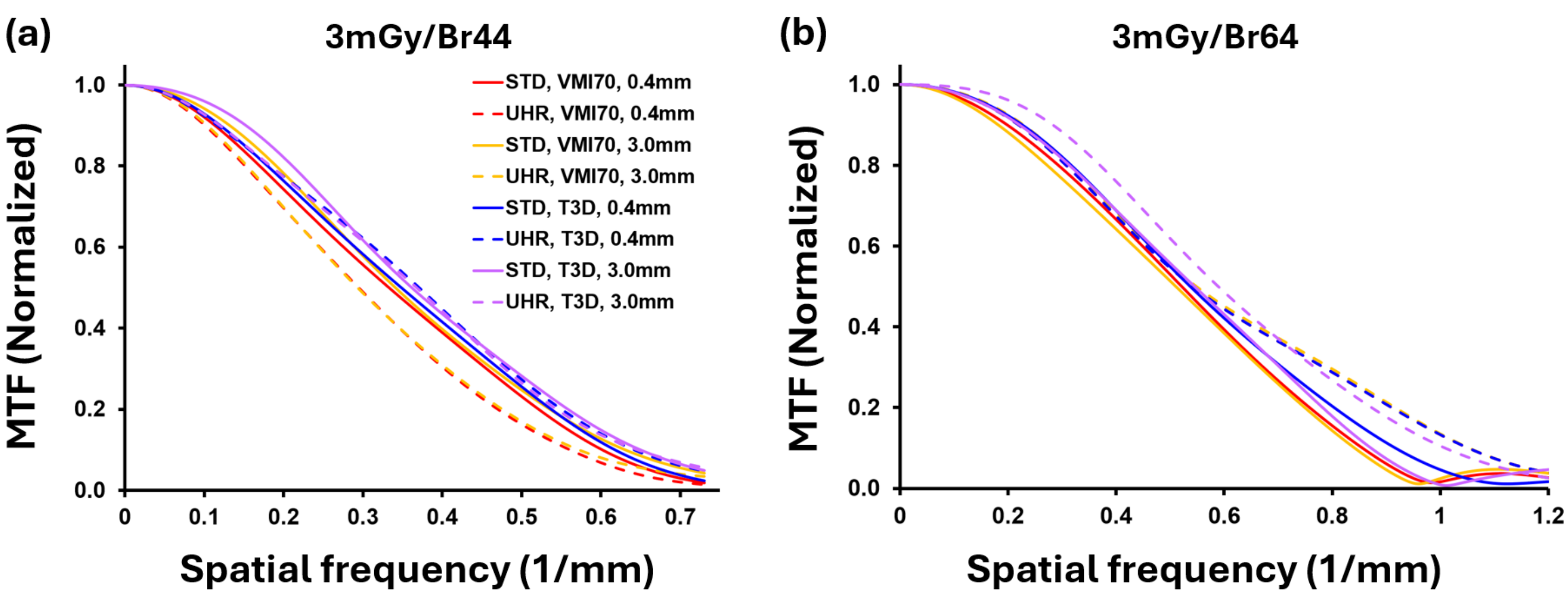

Figure S17. MTF for VMI (70 keV) and T3D reconstructions at 3 mGy for Br44 (a) and Br64 (b) kernels. Results are shown for STD and UHR modes at slice thicknesses of 0.4 mm and 3.0 mm.

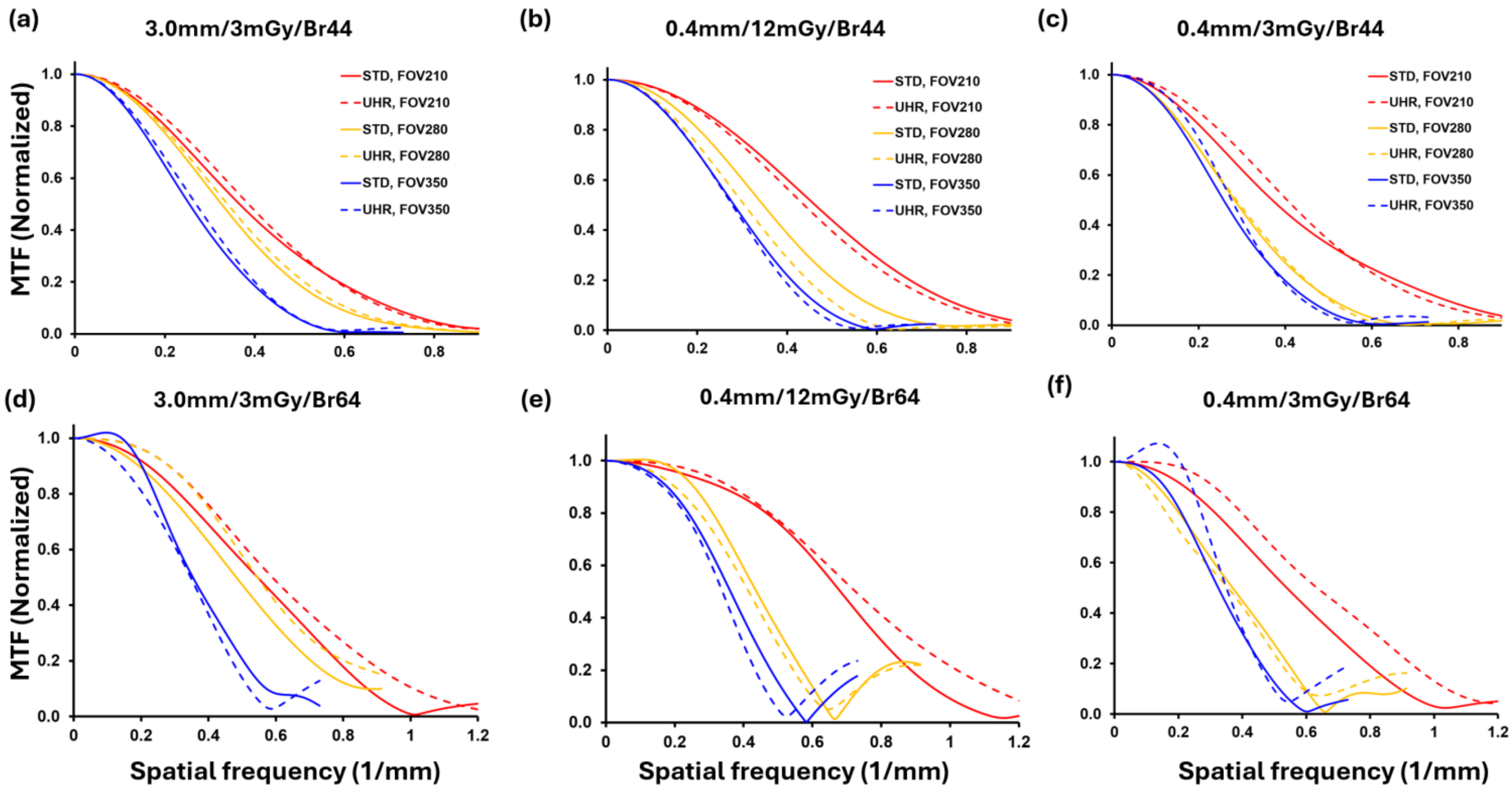

Figure S18. Effect of FOV on MTF for STD and UHR modes across multiple imaging conditions. Results are shown for Br44 (a–c) and Br64 (d–f) at different combinations of slice thickness and dose.
